# Humoral and cellular response in convalescent COVID-19 lupus patients

**DOI:** 10.1038/s41598-022-17334-5

**Published:** 2022-08-12

**Authors:** Cristina Solé, Sandra Domingo, Xavier Vidal, Josefina Cortés-Hernández

**Affiliations:** 1 Department of Rheumatology, Lupus Unit, Hospital Universitari Vall d´Hebron, Institut de Recerca (VHIR), Universitat Autònoma de Barcelona, Passeig Vall d´Hebron 119-129, 08035 Barcelona, Spain; 2 Clinical Pharmacology Service, Department of Pharmacology, Therapeutics and Toxicology, Fundació Institut Català de Farmacologia, Hospital Universitari Vall d’Hebron, Universitat Autònoma de Barcelona, 08035 Barcelona, Spain

**Keywords:** Translational immunology, Systemic lupus erythematosus

## Abstract

In SLE, underlying immune dysregulation and immunosuppression may increase the susceptibility to COVID-19 and impair the humoral and adaptive response. We aimed to characterize COVID-19 infection, identifying susceptibility and severity risk factors, assessing the presence of SARS-CoV-2 IgG antibodies and analyzing the cellular response. We established a prospective cohort of lupus patients to estimate the COVID-19 incidence compared to the reference general population. Data were collected via telephone interviews and medical record review. SARS-CoV-2 IgG antibodies were measured cross-sectionally as part of routine surveillance. Longitudinal changes in antibody titers and immunological profile from convalescent COVID-19 patients were evaluated at 6, 12 and 24 week after symptom onset. From immunological studies, PBMCs from convalescent patients were extracted and analyzed by flow cytometry and gene expression analysis. We included 725 patients, identifying 29 with PCR-confirmed COVID-19 infection and 16 with COVID-19-like symptoms without PCR-testing. Of the 29 confirmed cases, 7 had severe disease, 8 required hospital admission (27.6%), 4 intensive care, and 1 died. COVID-19 accumulated incidence was higher in lupus patients. Health care workers and anti-SSA/Ro52 antibody positivity were risk factors for COVID-19 susceptibility, and hypocomplementemia for severity. SARS-CoV-2 IgG antibodies were detected in 8.33% of patients. Three fourths of confirmed COVID-19 cases developed antibodies. High prednisone doses were associated with lack of antibody response. Antibody titers declined over time (39%). Convalescent patients at week 12 after symptom onset displayed a CD8^+^T cell reduction and predominant Th17 with a mild Th2 response, more pronounced in severe COVID-19 disease. Longitudinal immune response analysis showed a progressive sustained increase in CD8^+^ T and B memory cells with a decrease of Th17 signaling. Lupus patients are at higher risk of COVID-19 infection and new susceptibility and severity risk factors were identified. Lupus patients were able to mount humoral and cellular responses despite immunosuppressive therapy.

## Introduction

Severe acute respiratory syndrome coronavirus 2 (SARS-CoV-2), the causal agent of coronavirus disease 2019 (COVID-19), is a highly contagious virus associated with significant morbidity and mortality^1^. Despite drastic containment measures, the virus spread with a dramatic impact on public health. While some patients are asymptomatic, or have mild disease, others can develop severe symptoms including acute respiratory distress syndrome (ARDS) and cytokine storm, often leaving residual tissue damage, and death^2^. Proinflammatory cytokines, key mediators of late phase hyperinflammation, are induced by excessive innate immune activation due to delayed or impaired early immune response. Outcomes following COVID-19 infection vary considerably but older age, male gender, pre-existing comorbidities, immunologic abnormalities, and immunosuppression are consistent risk factors associated with poor prognosis^3^.

Systemic Lupus Erythematosus (SLE) is a chronic multisystem autoimmune disease characterized by a dysregulated innate and adaptative immune response^4^. Infections are common, being the second leading cause of death in SLE in developed countries. Therapeutic, disease-related, and genetic factors all contribute to increased susceptibility to infections which in turn, are potential triggers of disease flare^5^. Presumptively, SLE patients would be at risk of contracting SARS-CoV-2 and more likely to develop severe forms. While some studies indicate that lupus patients are not at risk^6–10^, other small case studies, mainly at the early phase of the pandemic, showed an increased risk of COVID-19 in SLE, although it is unclear whether they have a worse outcome^11–16^. Immune suppressive medications beyond steroids do not appear to increase the risk or severity of COVID-19. Results need to be read with circumspection as they can be influenced by methodological issues (e.g., exclusion of asymptomatic or mild disease), variable follow-up periods, limited availability or accuracy of PCR testing at the peak of the pandemic, and by the fact that SLE patients may have followed more strict protective behaviors to avoid exposure.

Innate and adaptative immunologic abnormalities prevalent in SLE may influence susceptibility to COVID-19. The increased type I interferon signature, characteristic of SLE, can confer a protective effect as first line antiviral defense^17^. Additionally, hydroxychloroquine (HCQ), a pivotal drug in SLE treatment, was initially considered potentially efficacious based on in vitro inhibition of viral replication as well as positive findings in murine models and limited clinical cases^18,19^. However, later studies failed to demonstrate significant clinical effectiveness^20,21^. On the other hand, inherent epigenetic dysregulation in lupus such as hypomethylation and overexpression of ACE2, a functional receptor for the SARS-CoV-2 spike glycoprotein, and demethylation of interferon-regulated key cytokine genes could facilitate viral entry, viremia, and an excessive immune response to SARS-CoV-2^22^.

The aim of this study was to determine the incidence of COVID-19 infection and to evaluate seroprevalence in a large cohort of lupus patients, as well as to provide insights of the underlying immune response to SARS-CoV-2 in convalescent patients.

## Methods

From March 15, right after lockdown, to September 15, 2020, we conducted a longitudinal telephone survey study at Vall d’Hebrón University Hospital Lupus Unit (Barcelona, Spain). Patients were identified from the database, telephonically interviewed by experienced trained professionals and their electronic medical notes extensively reviewed. After confinement we also performed a seroprevalence cross-sectional cohort study. Patients with PCR-confirmed COVID-19 infection had their serological and immunological response further evaluated. Data regarding the general population in Catalonia were retrieved from publicly available databases^23^. The study was in accordance with the Declaration of Helsinki and approved by the Vall D’Hebrón Ethics Committee (CIVIDSLE1). All participants provided written or oral informed consent.

### Study population

Eligible patients included adults (≥ 18 years) fulfilling the Systemic Lupus International Collaborating Clinics classification criteria (SLICC)^24^ or with Cutaneous Lupus Erythematosus (CLE) classified according to the 2004 Dusseldorf classification^25^. Inability to obtain informed consent was the exclusion criterion.

Participants responded monthly by telephone to a comprehensive questionnaire approved by the VHH review board, focused on COVID-19 symptoms, contact exposure and testing, comorbidities, demographic characteristics, lupus disease activity, damage and SLE medication (see supplementary questionnaire). For hospitalized or deceased patients, details of the hospital course were collected from their medical records. At inclusion the most recent (≤ 3 months) SLE Disease Activity index 2000 (SLEDAI-2 k)^26^ or CLASI score were recorded^27^. Active SLE disease was defined by a SLEDAI-2 k ≥ 6 or CLASI ≥ 4 in those with CLE. Damage was assessed by the Systemic Lupus International Collaborating Clinics/American College of Rheumatology (SLICC/ACR) Damage Index (SDI)^28^.

Following the European Centre for Disease Prevention and Control guidance^29^ patients with fever, dyspnea, persistent cough, or sudden onset of anosmia, ageusia or dysgeusia after February 2020, or radiological images compatible with COVID-19 were considered probable cases; confirmed cases were those with positive nasopharyngeal SARS-CoV-2 RT-PCR. Due to regional health policies, SARS-CoV-2 PCR testing was restricted to patients requiring hospital admission early in the pandemic. Only confirmed cases were used for comparison with the general population. COVID-19 disease was classified according to severity (see Supporting Information). From confirmed cases, additional clinical data, outcomes (recovered, improving, worsening or death) and sequelae at the time of analysis was obtained.

### Hydroxychloroquine dosage

Serum HCQ levels were measured using the Waters Alliance 2795 LC system and Micromass Quattro micro-API tandem mass spectrometry (positive ion, multiple reactions monitoring mode). A calibration curve (10–2.000 ng/mL) was generated to validate the method.

### Detection of SARS-CoV-2 antibodies

Between May 12 and August 15, 2020, after confinement, a cross-sectional seroprevalence study in available patients from the cohort (n = 300) was conducted. Patients testing positive for SARS-CoV-2 IgG antibodies were evaluated longitudinally at 6, 12 and 24 weeks after symptom onset. Two commercial assays for SARS-CoV-2 antibody testing were performed following manufacturer’s instructions: rapid test applied directly to finger-prick blood (Elabscience, UNCOV-4 COVID-19 IgG/IgM Rapid Test Cassette) and a chemiluminescent microparticle immunoassay (CLIA). An in-house ELISA assay was performed for antibody titration (see Supporting Information).

### Detection of anti-SSA/Ro52 antibodies

The determination of anti-SSA/Ro52 was performed by Hospital Vall Hebron Laboratories. They used a commercially available line immunoblot ENA assay (ANA Profile 3, Euroimmun AG, Lubeck, Germany). This kit provides a qualitative in vitro assay for human autoantibodies of the IgG class to 15 different antigens: nRNP/SM, Sm, SS-A, Ro-52, SS-B, Scl-70, PM-Scl, Jo-1, CENP B, PCNA, dsDNA, nucleosomes, histones, ribosomal P-protein and AMA M2 in serum or plasma. The results were evaluated using EUROLineScan software. For anti-Ro52 positive patients, value quantification used the ELISA test kit for human autoantibodies of the IgG class in serum or plasma following manufacturer’s instructions (anti-ENA Pool Elisa IgG, Euroimmun AG, Lubeck, Germany).

### Flow cytometry analysis (FACS)

PBMCs samples from COVID-19 convalescent patients were obtained (n = 12 at 6 and 24 weeks, and n = 20 at 12 weeks after symptoms onset). PBMCs from unexposed lupus patients were obtained as controls (n = 20). For cell surface staining, conjugated monoclonal antibodies were used (Table S1; see Supporting Information).

### Gene expression analysis

RNA from isolated PBMCs was obtained with RNeasy Mini Kit (Qiagen, Hilden, Germany) and cDNA with High-Capacity cDNA Reverse Transcription Kit (Applied Biosystems, Foster City, CA, USA). Gene expression was assessed by TaqMan gene expression assays (FAM dye labeled MGB probe, Applied Biosystems, Foster City, CA, USA) (Table S2; see Supporting Information).

### Cell culture

Isolated PBMCs from unexposed COVID-19 patients with or without anti-SSA/Ro52 antibodies (n = 5 in each group) were platted in a 24-well with complete RPMI media (RPMI, 10% FBS, 1% Pen/Strep, 2 mM/L-Glutamine) (Gibco, Life Technologies, Carlsbad, CA, USA). Cells were stimulated with RBD, S1 spike protein or PBS for 24 h (100 ng/well, Bioscience, Cambridge, UK). Afterwards, gene expression or immunofluorescence analyses were performed.

### Immunofluorescence

Cells were fixed and human anti-TRIM21 (1:250, Cloud-Clone Corp, Katy, TX, USA) was incubated at 4ºC overnight followed by a secondary Alexa-488-conjugated anti-rabbit IgG (1:500, Abcam, Cambridge, UK) (see Supporting Information).

### Statistical analysis

Descriptive statistics were used for the analysis of continuous variables expressed as mean (SD) and median with interquartile range (IQR), or range, when appropriate. Categorical variables were expressed as counts and proportion of patients (%). We compared continuous variables using t-test and ANOVA or Mann–Whitney and Kruskal–Wallis tests when appropriate, and categorial variables with Chi-squared or Fisher exact test. Multivariable logistic regression analyses were performed to control for potential confounding factors.

Standardized incidence rates and morbidity ratios were estimated using incidence data with all contemporaneous confirmed Covid-19 cases in Catalonia according to age and sex distribution. Data for Catalonian general population were obtained from the Conselleria de Salut, Generalitat de Catalunya^23^. For incidences and relative ratio results 95% confidence intervals were calculated. Longitudinal analyses were carried out using linear mixed models to deal with repeated measures. An exponential temporal covariance structure was used to account for the uneven time points. This approach allows for estimating differences between groups in each time point, as well as differences in change (“differences-in- differences") and the interaction between time and groups.

The statistical significance level was set at *p* < 0.05. All analyses were conducted using SAS 9.4 (SAS Institute Inc., Cary, NC, USA) statistical software.

### Ethics approval and consent to participate

The study was approved by the ethics committee of Vall Hebron Institute Research (CIVIDSLE1).

### Consent for publication

All participants in this study have consented for publication.

## Results

### Patient population and COVID-19 incidence

Overall, 758 patients were contacted, of whom 725 were enrolled between March 15 and April 1, 2020. Of those, we identified 29 with confirmed RT-PCR COVID-19, 16 with COVID-19-like symptoms who did not undergo confirmatory testing, and 680 without symptoms who were not tested (Table 1 and Table S3). The overall mean follow-up period was 5.90 months (range 0.07–6.08). Patients completed an average of 5 follow-up questionnaires^3–6^ throughout the study. Comparing the study groups there were more healthcare workers (HCWs) and patients with anti-SSA/Ro52 antibody positivity in the COVID-19 group. Number of patients receiving HCQ, treatment duration and serum HCQ concentration levels were similar between study groups (Table 1 and Figure S1).

**Table 1:** Baseline demographic and disease characteristics of the lupus patients classified by COVID-19 status*.

Characteristic	RT-qPCR confirmed COVID-19 infection (N = 29)	Non-COVID-19 patients (RT-qPCR not performed, N = 680)	*p*
Women	27 (93.10)	616 (90.59)	0.648
Age, mean (SD), years	50.52 (15.45)	49.36 (13.69)	0.657
Healthcare professional	9 (31.03)	35 (5.15)	< 0.001
**Race or ethnic group**			
White	24 (82.76)	588 (86.47)	0.569
Hispanic/Latin American Origen	5 (17.24)	92 (13.53)	
**Type of Lupus Disease**			
Cutaneous Lupus Erythematosus (CLE)	4 (13.79)	40 (5.88)	0.084
Systemic Lupus Erythematosus (SLE)	25 (86.20)	642 (94.41)	
**SLE disease activity**			
SLE disease, median (range), years	13.92 (1–31)	18.58 (1–50)	0.743
Total SLEDAI-2K score, range (SD)	1.55 (0–8)	1.89 (0–13)	0.147
SLEDAI ≥ 6	4 (13.79)	48 (7.09)	0.173
Overall SLICC-DI, median (IQR), years	0.41 (0–2)	0.32 (0–3)	1.000
Follow-up period	5.90 (0.07–6.08)	5.90 (0.07–6.08)	1.000
**History of SLE clinical features**			
Musculoskeletal	23 (79.31)	604 (88.82)	0.117
Mucocutaneous	22 (75.86)	518 (76.18)	0.969
Cardiorespiratory	9 (31.03)	128 (18.82)	0.103
Renal	8 (27.59)	37 (5.44)	< 0.001
Antiphospholipid syndrome	7 (20.69)	129 (18.97)	0.489
Hematological			
Leucopenia (< 3 × 10E9)	5 (17.24)	96 (14.11)	0.471
Lymphopenia (< 3 × 10E9)	10 (34.48)	149 (21.912)	0.112
**Autoantibody status**			
ANA titer ≥ 1/80	28 (96.55)	591 (86.91)	0.127
Anti-dsDNA antibodies ≥ 15 IU/mL	9 (31.03)	252 (37.05)	0.510
Anti-dsDNA antibodies, mean (range), IU/mL	74.02 (2–626)	66.21 (3–225)	0.543
C3 and/or C4 below lower limit of normal	6 (20.69)	204 (30.00)	0.282
Anti-SSA/Ro antibodies	12 (41.38)	97 (14.26)	< 0.001
Anti-SSB/La antibodies	3 (10.34)	35 (5.14)	0.224
Anti-RNP antibodies	3 (10.34)	25 (3.68)	0.0710
Anti-Sm antibodies	2 (6.89)	18 (2.65)	0.176
**Coexisting comorbidities**			
Hypertension	12 (41.38)	235 (34.55)	0.450
Cardiovascular disease	3 (9.38)	54 (7.94)	0.641
Pulmonary disease	1 (3.45)	40 (5.88)	0.582
Diabetes	2 (6.90)	26 (3.82)	0.405
Obesity	2 (6.90)	57 (8.38)	0.777
Depressive syndrome	1 (3.45)	62 (9.12)	0.293
**COVID-19**			
Known COVID-19 contact	20 (68.96)	0 (0.00)	na
**COVID-19 symptoms**			
No symptoms	0 (0)	680 (100)	na
Fever	26 (89.65)	0 (0)	na
Shortness of breath	13 (44.83)	0 (0)	na
Cough	24 (82.75)	0 (0)	na
Anosmia/Ageusia	22 (75.86)	0 (0)	na
Diarrhea	8 (27.59)	0 (0)	na
Fatigue	16 (55.17)	0 (0)	na
Headache	15 (51.72)	0 (0)	na
Treatments			
Antimalarial agents	14 (48.28)	342 (50.29)	0.831
Serum concentration levels, mean (range), ng/mL	1247 (550–4500)	1194 (577–2983)	0.758
**Immunosuppressive therapy**	21 (72.41)	349 (51.32)	0.026
MMF/EC-MPS	12 (41.38)	190 (27.94)	0.116
Azathioprine	4 (13.79)	61 (8.97)	0.378
Tacrolimus	8 (27.59)	113 (16.62)	0.124
Methotrexate/leflunomide	2 (6.90)	59 (8.67)	0.738
**Biological therapy**	4 (13.79)	63 (9.26)	0.414
Tocilizumab	1 (3.45)	7 (10.29)	0.227
Etanercept	3 (10.34)	38 (11.76)	0.282
Belimumab	0 (0.00)	15 (22.06)	0.419
Rituximab	0 (0.00)	3 (0.44)	0.720
**Corticosteroids use**,	18 (62.07)	448 (65.88)	0.672
Daily prednisone dose, mean (range), mg/day	4.07 (0–15)	3.45 (0–40)	0.282
> 7.5 mg/day at baseline	5 (17.24)	35 (5.15)	0.006

*Except where indicated otherwise, values are the number (%).

*RT-qPCR* Reverse-transcriptase quantitative PCR; *SLE* Systemic lupus erythematosus; *CLE* Cutaneous Lupus Erythematosus; *SLEDAI-2K* Systemic Lupus Erythematosus Disease Activity Index 2000; *SLICC-DI* Systemic Lupus International Collaborating Clinics Damage Index. *MMF* mycophenolate mofetil; *EC-MPS* enteric-coated mycophenolate sodium; *na* not applicable. Reference ranges are as follows: anti-double-stranded DNA antibodies, < 15 IU per milliliter; serum C3 (mg/dL), 85 to 110; serum C4 (mg/dL), 10 to 40.

**Figure 1:**
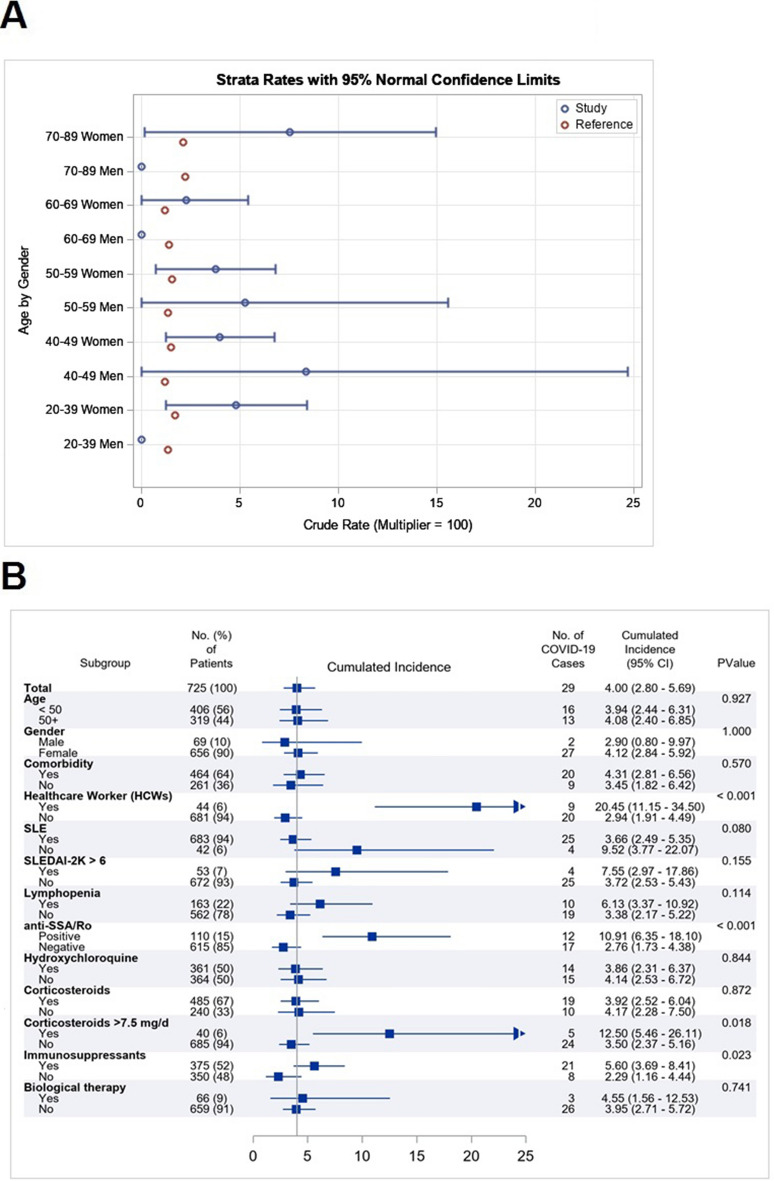
Covid-19 incidence in Lupus Disease. (**A**) Age and gender-stratified COVID-19 incidence estimates in lupus patients (blue), compared with reference population (red). (**B**) Forest plot of COVID-19 cumulated incidence according to several subgroups of interest.

Statistical data of confirmed COVID-19 incidence in Catalonia during the same period was 1.55% and included only patients aged 20 to 89 years-old. Selecting this age range from the cohort (n = 713) showed a higher incidence of confirmed COVID-19 infection in lupus patients (29 out 713 lupus (4.07%, 95% CI 2.85–5.78). After indirect standardization, the estimated incidence was remarkably similar (4.05%, 95% CI 2.58–5.52), with a standardized morbidity ratio of 2.61 ((95% CI 1.66–3.56), *p* < 0.001). Eliminating health professionals as a possible confounding factor still left an increased estimated incidence of 2.98 (1.67–4.28). This increment was observed across all ages with no differences between sexes (Fig. 1A).

### Clinical characteristics and outcomes of RT-PCR confirmed COVID-19 patients

Patient baseline characteristics are shown in Table 1. There was a female predominance (93.1%), mean age 50.52 years (range 18–90) with systemic disease (86.20%). The most common COVID-19 symptoms were fever (89.65%) and cough (82.75%). Eleven patients (37.93%) developed bilateral pneumonia. Patients with COVID-19 received higher prednisone doses (≥ 7.5 mg/day) (17.24%) and immunosuppressive treatment (72.41%), mycophenolate mofetil (MMF) being most common (41.38%). There was no increased lupus disease activity during COVID-19 infection (Table 1).

Most patients had mild COVID-19 disease (75.86%), but seven (24.14%) progressed to a severe form (Table 2). Eight patients (27.59%) required hospital admission, of whom 5 developed acute respiratory distress syndrome, 4 were admitted into ICU and one died. The accumulated incidence of ICU admission in the general population was 0.86%. Intensive care admissions were higher in lupus patients (4 out 29, 13.79%, *p* < 0.001). The median duration of ICU stay was 8 days (IQR 8–13 days). All patients with severe COVID-19 disease had SLE. Severe COVID-19 cases developed more bilateral ground-glass pneumonia and had higher serum ferritin and IL-6 levels (Table 2). However, the strongest association with severe COVID-19 was hypocomplementemia (RR = 6.40, 95% CI 2.04–20.12, *P* = 0.007).

**Table 2:** Severe and mild RT-qPCR confirmed COVID-19 lupus patients*.

Characteristic	Severe COVID-19 (N = 7)	Mild COVID-19 (N = 22)	*p*
Women	6 (85.71)	21 (95.45)	0.431
Age, mean (SD), years	52.57 (14.07)	49.32 (15.85)	0.632
Healthcare professional	0 (0)	9 (40.91)	0.066
**Race or ethnic group**			
White	5 (71.42)	19 (86.36)	0.569
Hispanic/Latin American Origen	2 (28.57)	3 (13.64)	0.569
**Type of Lupus Disease**			
Cutaneous Lupus Erythematosus (CLE)	0 (0)	4 (18.18)	0.546
Systemic Lupus Erythematosus (SLE)	7 (100)	18 (81.81)	0.546
**SLE disease activity**			
Total SLEDAI-2K score, range (SD)	2.57 (0–6)	1.23 (0–8)	0.138
SLEDAI ≥ 6	1 (14.28)	3 (13.64)	1.000
Overall SLICC-DI, median (IQR), years	0.42 (0–2)	0.41 (0–1)	0.932
**History of SLE clinical features**			
Musculoskeletal	7 (100)	16 (72.73)	0.289
Mucocutaneous	5 (71.43)	17 (77.27)	0.558
Cardiorespiratory	3 (42.86)	6 (27.27)	0.642
Renal	3 (42.86)	5 (22.73)	0.357
Hematological	1 (14.29)	6 (27.27)	0.646
Leucopenia (< 3 × 10E9)	1 (14.29)	4 (18.18)	1.000
Lymphopenia (< 3 × 10E9)	2 (28.57)	8 (36.36)	1.000
Autoantibody status			
Anti-dsDNA antibodies ≥ 15 IU/mL	3 (42.86)	6 (27.27)	0.642
Anti-dsDNA antibodies, mean (range), IU/mL	166 (9–626.70)	188.87 (5–633)	0.821
C3 and/or C4 below lower limit of normal	4 (57.14)	2 (9.09)	0.018
Anti-SSA/Ro antibodies	2 (28.60)	10 (45.45)	0.665
Anti-SSB/La antibodies	1 (14.29)	3 (13.64)	1.000
**Coexisting comorbidities, n (%)**			
Hypertension	3 (42.85)	9 (40.91)	1.000
Cardiovascular disease	0 (0.00)	3 (13.63)	0.558
Pulmonary disease	0 (0.00)	1 (4.54)	1.000
Diabetes	1 (14.29)	1 (4.54)	0.431
Obesity	0 (0.00)	2 (9.09)	1.000
Depressive syndrome	0 (0.00)	1 (4.54)	1.000
**Treatments**			
Antimalarial agents	3 (42.86)	11 (50.00)	1.000
**Immunosuppressive therapy**	7 (100)	14 (63.64)	0.142
MMF/EC-MPS	4 (57.14)	8 (36.36)	0.403
Azathioprine	1 (14.29)	3 (13.64)	1.000
Tacrolimus	3 (42.86)	5 (22.73)	0.354
Methotrexate/leflunomide	0 (0)	2 (9.09)	1.000
**Biological therapy**	1 (14.28)	3 (13.64)	1.000
Tocilizumab	0 (0)	1 (4.54)	1.000
Etanercept	1 (14.28)	2 (9.09)	1.000
**Corticosteroids use**	4 (57.14)	14 (63.63)	1.000
Daily prednisone dose, mean (SD), mg/day	2.14 (2.36)	1.63 (2.63)	0.623
> 7.5 mg/d at baseline	0 (0)	5 (22.73)	0.297
**COVID-19 symptoms**			
Fever	7 (100)	19 (86.36)	0.558
Shortness of breath	6 (85.71)	7 (31.82)	0.026
Cough	6 (85.71)	18 (81.82)	1.000
Anosmia/Ageusia	6 (85.71)	16 (72.73)	0.646
Diarrhea	1 (14.29)	7 (31.82)	0.635
Fatigue	5 (71.43)	11 (50.00)	0.410
Headache	6 (85.71)	9 (40.91)	0.080
HRCT Bilateral Pneumonia	7 (100)	4 (18.18)	< 0.001
Hospital admission	7 (100)	1 (4.55)	< 0.001
ICU admission	4 (57.14)	0 (0)	< 0.001
Exitus	1 (14.29)	0 (0)	0.241
**Laboratory parameters during COVID-19 infection**			
Ferritin, mean (range), mg/dL	646.71 (50–1758)	122.50 (47–348)	< 0.001
Leucocytes, mean (range)	9.07 (3.4–17.19)	5.96 (4.14–9.15)	0.012
Lymphocytes, mean (range)	1.19 (0.70–2.00)	1.41 (0.80–2.00)	0.259
D-dimer, mean (range)	870.50 (87–2795)	1106 (50–3852)	0.724
LDH, mean (range)	371.50 (283–514)	318.60 (182–595)	0.440
PCR, mean (range)	13.58 (0.70–27.71)	7.01 (0.06–21.60)	0.141
IL6, mean (range)	96.27 (4.50–223.00)	3.28 (1.50–5.60)	< 0.001

*Except where indicated otherwise, values are the number (%).

*RT-qPCR* Reverse-transcriptase quantitative PCR; *SLE* Systemic lupus erythematosus; *CLE* Cutaneous Lupus Erythematosus; *SLEDAI-2K* Systemic Lupus Erythematosus Disease Activity Index 2000; *SLICC-DI* Systemic Lupus International Collaborating Clinics Damage Index. *MMF* mycophenolate mofetil; *EC-MPS* enteric-coated mycophenolate sodium. Reference ranges are as follows: anti-double-stranded DNA antibodies, < 15 IU per milliliter; serum C3 (mg/dL), 85 to 110; serum C4 (mg/dL), 10 to 40.

After 6 months follow-up, three patients with pneumonia (27.3%) had not recovered the pulmonary opacities, but lung function test progressively improved. Three thrombotic events were recorded (10.34%) including two pulmonary embolisms, and one mesenteric thrombosis. Median duration to thrombotic event post discharge was 73 days (interquartile range, 12–150). All received prophylactic low-molecular-weight heparin up to 15 days post-discharge. None had antiphospholipid antibodies.

### Exploratory risk subgroup analyses for COVID 19 infection in SLE

Although the subgroups were small, HCWs (20.45% vs. 2.94%, *p* < 0.001), those with anti-SSA/Ro52 antibodies (10.91% vs. 2.76%, *p* < 0.001), higher prednisone doses (≥ 7.5 mg/day) (12.50% vs 3.50%, *p* = 0.018), or immunosuppressant treatment (5.60% vs 2.29%, *p* = 0.023) developed more COVID-19 infection (Fig. 1B). The relative risk was 6.96 (95% CI 3.37–14.38) for HCWs, 3.95 (95% CI 1.94–8.03) for anti-SSA/Ro52 positivity, 3.57 (95% CI 1.44–8.86) for prednisone doses ≥ 7.5 mg/day, and 2.45 (95% CI 1.10–5.46) for immunosuppressant therapy. These values did not change substantially when a multivariable analysis was performed (Table S4). These risk factors did not influence disease severity. Classic comorbidity variables were not found different amongst the study groups.

### TRIM21 is reduced in COVID-19 lupus patients with anti-SSA/Ro52 positive antibodies

**Figure 2:**
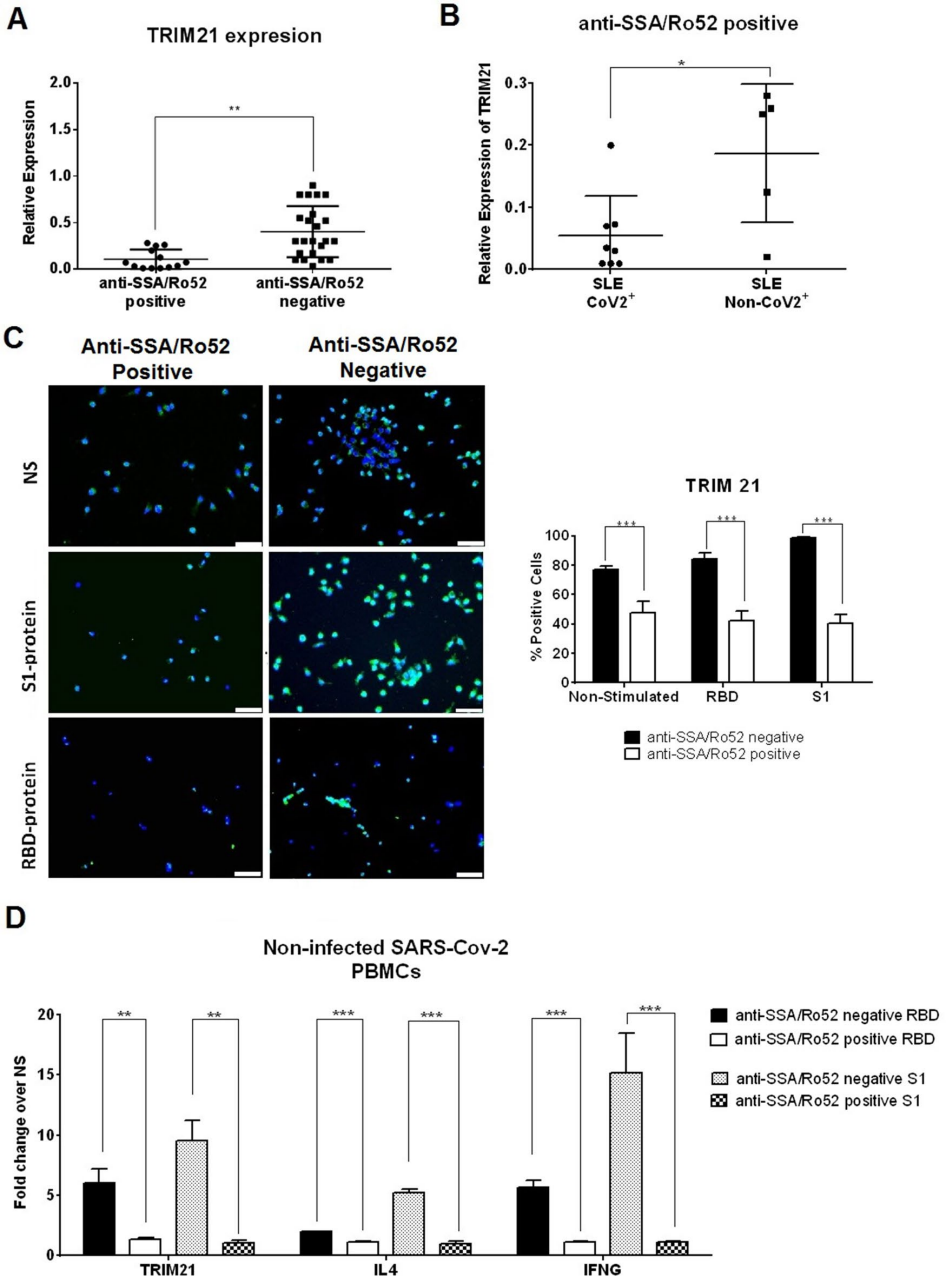
TRIM21 gene expression and protein levels in peripheral blood mononuclear cells (PBMCs) from lupus patients in the presence or absence of anti-SSA/Ro52 antibodies (N = 45). (**A**) TRIM21 expression levels in PBMCs obtained from unexposed lupus patients with anti-SSA/Ro52 (positive, N = 13) and without anti-SSA/Ro52 (negative, N = 32) antibodies. Relative gene expression was calculated using the 2^−ΔΔCt^ method and GADPH was used as endogenous control. **p < 0.005. (**B**) Quantitative real-time reverse-transcriptase PCR (RT-qPCR) of TRIM21 in lupus patients with anti-SSA/Ro52 positive antibodies exposed to SARS-CoV-2 (CoV2 +) or unexposed (Non-CoV2 +). *p < 0.05. (**C**) PBMCs from unexposed lupus patients with or without anti-SSA/Ro52 antibodies were stimulated with RBD protein, S1 protein or NS (non-stimulated condition, only PBS stimulation). After 24 h, PBMCs were analyzed by immunofluorescence to measure TRIM21 protein expression levels (green). Blue color (DAPI) labels cell nuclei. Error bars represent the mean ± SEM from three independent experiments. Scale bar = 50 µm. ***p < 0.0001. (**D**) TRIM21, IL4 and INFG gene expression was evaluated in PBMCs from unexposed SARS-CoV-2 lupus patients after RBD or S1 protein stimulation. Patients were subdivided into two groups according to the presence or absence of anti-SSA/Ro52 antibodies. In vitro stimulation was performed using RBD or S1 protein over 24 h. RT-qPCR gene expression analysis was performed thereafter. Fold change in expression level was calculated over non-stimulated conditions (PBS stimulation). **p < 0.005, ***p < 0.0001. All *p*-values were obtained using unpaired t-test.

Under viral infection, Ro52 (so called TRIM21) is upregulated through the IFN/JAK/STAT signaling pathway, promoting innate immune response. TRIM21 gene expression levels in PBMCs from unexposed lupus patients were lower in those with anti-SSA/Ro52 positive antibodies compared with negatives (0.106 vs 0.410, *p* = 0.004, Fig. 2A). There was a correlation between TRIM21 expression and anti-SSA/Ro52 titers (r =  − 0.379, *p* = 0.0251, Figure S2). TRIM21 levels decreased further in anti-SSA/Ro52 positive patients with COVID-19 infection (0.055 vs 0.187, *p* = 0.018, Fig. 2B). In vitro PBMCs stimulation confirmed the TRIM21 reduction (Fig. 2C) and also showed reduced *IL4* and *INFG* expression (Fig. 2D). No changes were observed in type I IFN or Th17 signaling pathways (Figure S3).

### Seroprevalence of anti- SARS-CoV-2 antibodies

**Figure 3:**
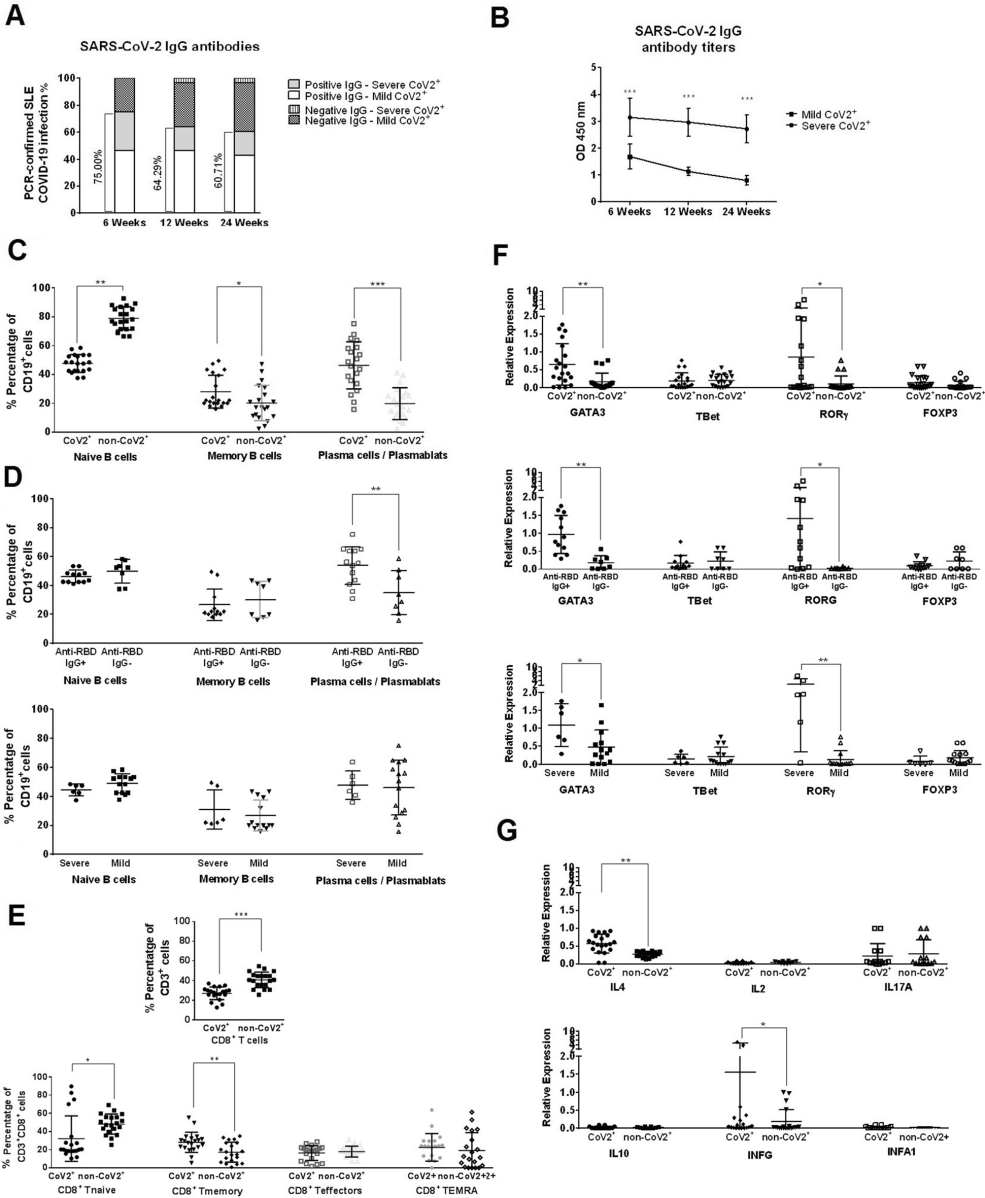
SARS-CoV-2 IgG antibodies and cellular profile in COVID-19 convalescent lupus patients. (**A**) Proportion of positive or negative SARS-CoV-2 IgG antibodies in the 29 lupus patients with confirmed COVID-19 at 6, 12 and 24 weeks after onset of symptoms. Lupus patients were classified according their COVID-19 severity (mild and severe CoV2 +). (**B**) SARS-CoV-2 IgG titers in COVID-19 lupus samples according to disease severity (severe vs mild) at 6, 12 and 24 weeks after onset of symptoms. Optical density (O.D.) was measured at 450 nm. Data is represented as mean ± SEM. *p* value was obtained using paired t-test. ***p < 0.0001. (**C**) Proportion of CD19^+^IgD^−^CD27^−^ (naïve B cells), CD19^+^IgD^+^CD27^+^ (memory B cells) or CD19^+^CD27^+^CD38^+^ (plasma cells / plasmablasts) obtained by flow cytometry between lupus COVID-19 and non-COVID-19 patients (CoV2^+^ and non-CoV2^+^). : *p < 0.05, **p < 0.005, ***p < 0.0001. (**D**) RT-PCR COVID-19-confirmed lupus patients were classified according to presence or absence of SARS-CoV-2 IgG antibodies to RBD protein (anti-RBD IgG^+^ or IgG^−^) or by disease severity (severe or mild). Percentage of naïve, memory B cells and plasma cells/plasmablasts were analyzed at 12 weeks after onset of symptoms. **p < 0.005. (**E**) Representative flow cytometry plots of CD8^+^ total cells (CD3^+^CD8^+^), CD8^+^ naïve cells (CD3^+^CD8^+^CD45RA^+^CCR7^+^), CD8^+^ memory cells (CD3^+^CD8^+^CD45RA^−^CCR7^+^),CD8^+^ effector cells (CD3^+^CD8^+^CD45RA^−^CCR7^−^) and TEMRA CD8^+^ cells (CD3^+^CD8^+^CD45RA^−^CCR7^−^) from COVID-19 confirmed patients vs. unexposed lupus patients. *p < 0.05, ***p < 0.0001. (**F**) Relative expression levels of transcription factors of Th1 (GATA3), Th2 (TBet), Th17 (RORG) and Treg (FOXP3) subsets in exposed and un-exposed lupus patients (CoV2^+^ and non-CoV2^+^). Gene expression was also studied according to SARS-CoV-2 antibody production (anti-RBD IgG^+^ or IgG^−^) and COVID-19 disease severity (severe or mild). Relative gene expression was calculated using the 2-ΔΔCt method and GADPH was used as endogenous control. *p < 0.05, **p < 0.005. (**G**) Interleukin production was measured by RT-qPCR gene expression analysis in exposed and unexposed lupus patients (CoV2^+^ and non-CoV2^+^). *p < 0.05, **p < 0.005. *p*-values are obtained using t-test in (**C**) to (**G**).

After confinement we performed a cross-sectional serological evaluation to detect past infection and assess individual antiviral humoral responses. The first measurement was obtained at a mean of 6 weeks after the onset of symptoms (range, 5 to 8), and the last at 24 weeks (range, 21 to 25). Twenty-five (8.33%) of the 300 patients evaluated had a positive SARS-CoV-2 IgG antibody test. Antibodies were found by week 6 in 21 of the 28 (75%, Fig. 3A) available patients with RT-PCR confirmed COVID-19 and in 2 patients of the 16 (12.5%) who had COVID-19-related symptoms without RT-PCR testing. The remaining 2 patients who developed an antibody response were asymptomatic (2 out of 256, 0.78%). To identify factors associated with the antibody response, we compared SARS-CoV-2 antibody-positive and negative patients amongst the PCR-confirmed COVID-19 group. Prednisone ≥ 7.5 mg/day was associated with lack of antibody response (*p* = 0.038). Furthermore, the seronegative group received more MMF (71.43% *vs* 28.57%) (*p* = 0.076) (Table S5).

Longitudinal serial testing of patients with RT-PCR confirmed COVID-19 (n = 28) showed a reduction in the antibody titers (Fig. 3A). Seven patients had not presence SARS-CoV-2 IgG positivity at week 6 and four lost their positivity at week 24. The patients who lost the positivity for SARS-CoV-2 IgG titers were patients receiving immunosuppressive therapy (50% with MMF). This reduction was more pronounced in patients with mild COVID-19 disease (Fig. 3B). Clinically, they had more articular and hematological manifestations (75% of them). Immunologically, no significantly changes were found in T cells, B cells or plasma cells / plasmablasts.

### Cellular immune response to SARS-Cov-2 in COVID-19 convalescent lupus patients

PBMCs from COVID-19 convalescent patients at week 12 were analyzed by flow cytometry (n = 6 severe, n = 14 mild) and compared with unexposed lupus patients. Characterization of the B cell response showed no changes in CD19 + B cells with a decreased percentage of CD19 + CD27-IgD + naive B cells (47.6% vs 78.9%, *p* < 0.001), an increased frequency of CD19 + CD27 + IgD- memory B cells (28.1% vs. 20.2%, *p* = 0.043) and CD19 + CD38 + CD27 + plasma cells / plasmablasts (46.4% vs. 19.8%, *p* < 0.001) (Fig. 3C and Figure S4). The increment was higher in patients with higher IgG antibody titers (53.7% vs. 35.0%, *p* = 0.008). There were no differences according to disease severity (Fig. 3D).

There was also a disrupted T cell homeostasis with reduced CD8^+^T cell percentage (26.9% vs 40.6%, *p* < 0.001, Fig. 3E and Figure S4), especially of naïve CD8^+^T cells (32.24% vs. 48.21%, *p* = 0.013) compared to unexposed patients. Increased memory CD8^+^T cells were noted (28.09% vs. 17.28%, *p* = 0.0037, Fig. 3E), more pronounced in severe disease (Figure S5). No differences were observed in total, memory, naïve, effector and terminal differentiated effector memory (TEMRA) CD4^+^T cells, NK cells or cell activation (Figure S6). Among CD4^+^ T cells, there was a predominant Th2 and Th17 response with increased gene expression of *GATA3* and *RORG* transcription factors, more prominent in severe COVID-19 and SARS-CoV-2 antibody-producing patients (Fig. 3F). Measurement of proinflammatory cytokines showed only increased *IL4* and *IFNG* expression levels. There were no changes in IFN-induced genes (Fig. 3G and Figure S7).

### Longitudinal immune dynamics of COVID-19 convalescent patients

**Figure 4:**
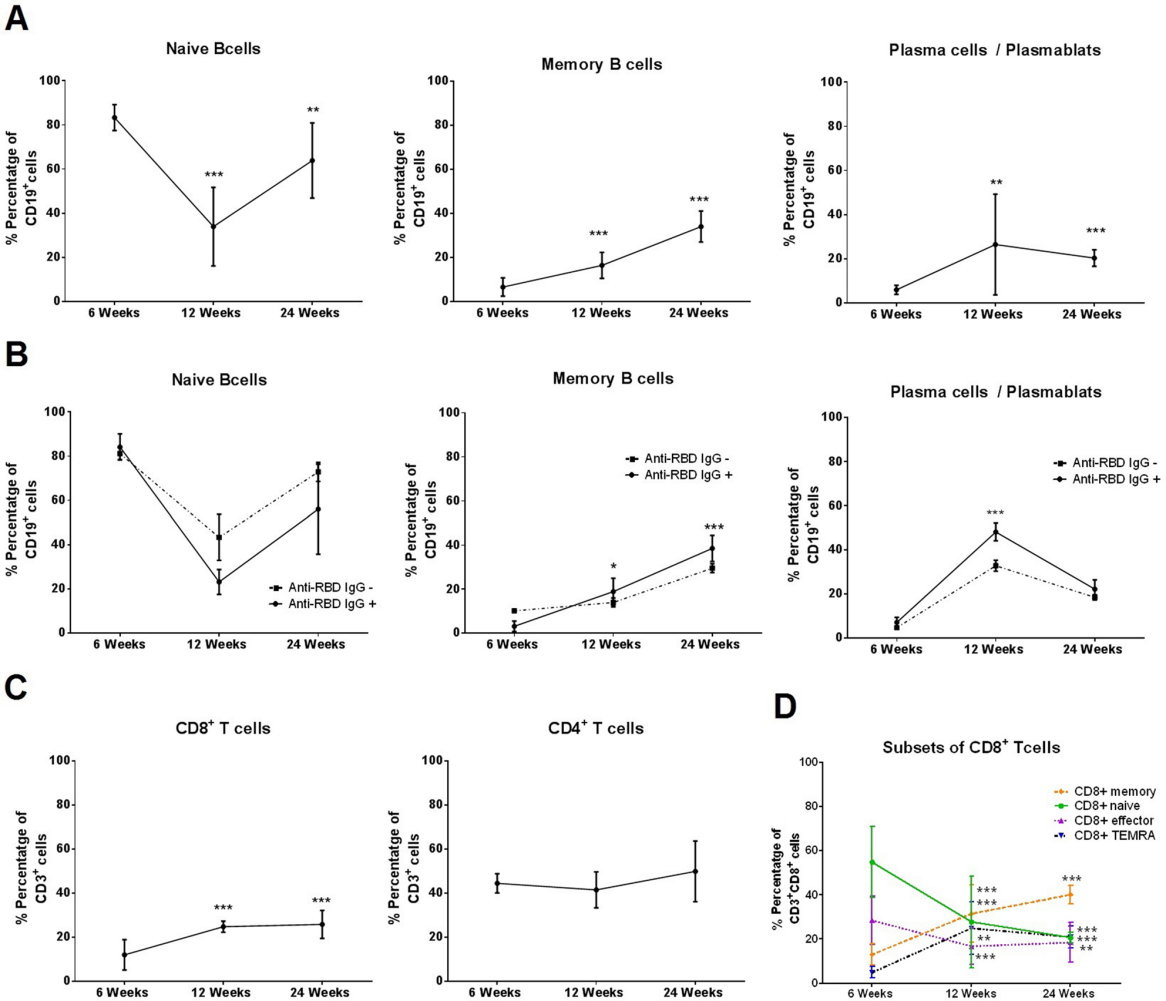
Longitudinal cellular profile revealed an enrichment of memory B and CD8^+^ T cell in exposed SARS-CoV-2 lupus patients. (**A**) Longitudinal study of the percentage of CD19^+^IgD^−^CD27^−^ (naïve B cells), CD19^+^IgD^+^CD27^+^ (memory B cells) or CD19^+^CD27^+^CD38^+^ (plasma cells / plasmablasts) at 6, 12 and 24 weeks after onset of symptoms in SARS-CoV-2 exposed lupus patients (N = 12). Significant differences were calculated in comparison with the 6-week timepoint using paired t-test. *p < 0.05, **p < 0.005, ***p < 0.0001. (**B**) Patients who produced SARS-CoV-2 antibodies against RBD protein (anti-RBD IgG^+^) have significantly increased levels of memory B cells at Week 12 and 24, and antibody-producing cells (plasma cells / plasmablasts) at Week 12 compared with anti-RBD IgG^−^ patients. No differences were found in naïve B cells. Fold change significant differences over time were calculated between groups by linear mixed models. *P < 0.05, ***p < 0.001. (**C**) By flow cytometry, CD3^+^ CD8^+^ T cell percentages were significantly increased after 6 weeks. No differences were found in CD3^+^ CD4^+^ T cells. *p*-values were obtained using paired t-test . *p < 0.05, **p < 0.005, ***p < 0.0001. (**D**) Longitudinal dynamics of CD8^+^ T cell subtypes in convalescent SARS-CoV-2 lupus patients at 6-, 12- and 24-weeks post symptom onset. The vertical axis shows the percentage of CD3^+^ CD8^+^ cells, and the horizontal axis shows time. Error bars represent the mean ± SEM from 12 lupus patients. Significant differences were calculated in comparison with the 6-week timepoint using paired t-test. **p < 0.005, ***p < 0.0001.

Twelve convalescent patients’ cellular immune responses were studied longitudinally (Table S6). A transient increase in the percentage of plasma cells / plasmablasts and a reduction of naïve B cells (83.3 vs 33.9%, *p* < 0.001) were apparent at 12 weeks (*p* < 0.001) reverting by week 24. Meanwhile, memory B cells showed a progressive increase over time (*p* = 0.01, Fig. 4A). These changes were more pronounced in antibody-producing vs non-antibody producing patients: for plasma cells / plasmablasts a difference in the increase of 13% at 12 week (*p* < 0.001) and for memory B cells an increase of 12.0% and 16.0% at 12 and 24 week (*p* = 0.006 and *p* < 0.001, respectively. Figure 4B). B cell response was not affected by disease severity (Figure S8).

**Figure 5:**
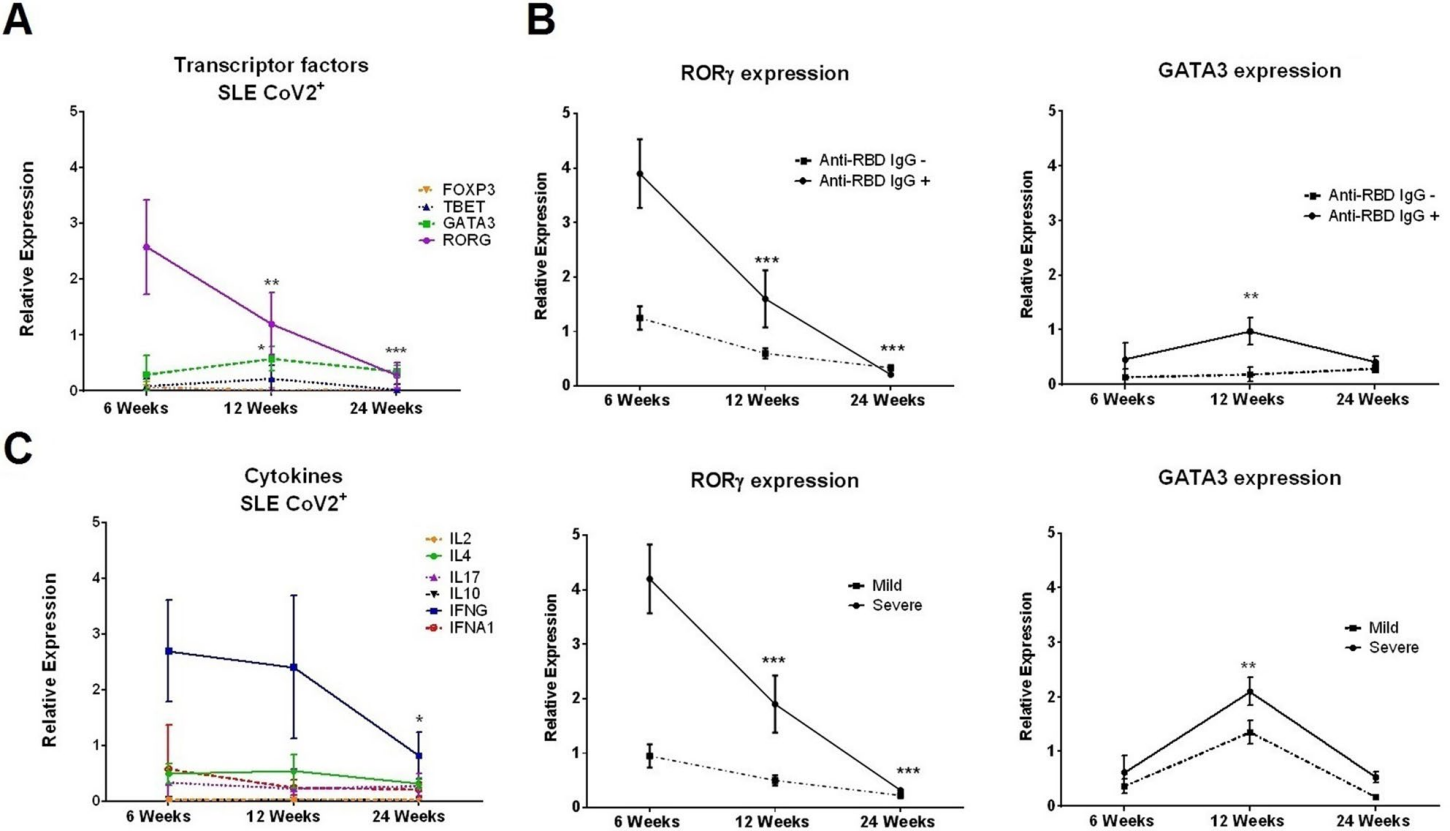
A decrease of Th17 signaling was observed in immune longitudinal analysis in exposed SARS-CoV-2 lupus patients. (**A**) Gene expression of transcription factors at 6-, 12- and 24-weeks post symptom onset (N = 12). The vertical axis shows the relative expression, and the horizontal axis shows time. Error bars represent the mean ± SEM from 12 lupus patients. Significant differences were calculated in comparison with the 6-week timepoint using paired t-test. *p < 0.05, ***p < 0.0001. (**B**) Gene expression of transcription factors for Th17 (RORG) and Th2 (GATA3) were evaluated longitudinally in convalescent SARS-CoV-2 lupus patients according to the production of antibodies against RBD protein (anti-RBD IgG^+^ or IgG^−^) and disease severity (mild or severe). Fold change significant differences over time were calculated between groups by linear mixed models *p < 0.005, ***p < 0.001. (**C**) Cytokines were evaluated longitudinally in convalescent lupus patients by RT-qPCR gene expression analysis. Significant differences were calculated in comparison to the 6-week timepoint. Only interferon gamma (INFG) was significantly decreased at 24 weeks using paired t-test. *p < 0.05.

CD8^+^ T cell total numbers recovered progressively after week 6 (*p* < 0.001) but no changes were observed in CD4^+^ T cells (Fig. 4C). There was a reduction of naïve and effector CD8^+^ T cells and a progressive sustained increase in memory and TEMRA CD8^+^ T cells during the study period, regardless of disease severity (Fig. 4D and Figure S9). Of the transcription factors tested, only *RORG* gene expression levels were significantly high at week 6, progressively reducing thereafter (*p* = 0.001, Fig. 5A). Higher *RORG* expression was associated with poor outcome (persistent lung opacities (n = 2) and thrombosis (n = 3)) (Figure S10 and Table S7). *GATA3* levels showed a mild increase by week 12 (*p* = 0.022, Fig. 5A). All changes were more pronounced in antibody-producing vs non-producing patients: for *RORG* gene expression a decrease of 1.6 and 2.8 at 12 weeks and 24 weeks (*p* < 0.001) and for *GATA3* gene expression an increase of 0.5 at 12 week (*p* = 0.005). In those with severe vs mild disease: for *RORG* gene expression an increase of 1.9 and 3.1 at 12 and 24 weeks (*p* < 0.001) and for *GATA3* gene expression a decrease of 0.6 at 12 weeks (*p* = 0.001, Fig. 5B). No significant changes were observed in *Tbet* and *FOXP3* expression (Fig. 5A). Of the cytokines tested, only *IFNG* expression levels were significantly raised at weeks 6 and 12 reducing by week 24 (*p* < 0.001) regardless of antibody production or disease severity (Fig. 5C and Figure S11).

## Discussion

This study represents one of the largest cohorts of lupus patients from the early phases of the COVID-19 pandemic, in which we evaluate the risk of infection and characterize SARS-CoV-2-specific humoral and cellular immunity in PCR-confirmed COVID-19 patients.

Several early studies sought to describe COVID-19 epidemiology in lupus. Using clinical registries, patients’ surveys, and reviews of hospital records, studies also faced certain limitations like the exclusion of asymptomatic or mild cases, or PCR test accuracy and availability. Whereas many later studies showed that lupus patients were not at increased risk of COVID-19^6–10^, early small case studies suggested higher risk and worse outcomes^11–16^. We identified 29 lupus patients with RT-PCR-confirmed COVID-19 representing an elevated accumulated incidence of the disease compared with the general population (4.07% vs 1.55%).

In agreement with previous studies, we showed high doses of corticosteroids and immunosuppressive therapy to be risk factors for SARS-CoV2 infection and disease severity^30^. In our cohort other classic comorbidities described in the general population were not found to be predictive^31–34^. Our data supports the ineffectiveness of hydroxychloroquine to prevent COVID-19 disease^21,22^ with similar rates of COVID-19 in hydroxychloroquine-treated and untreated patients. We have also identified HCWs and anti-SSA/Ro52 antibody positivity as risk factors for COVID-19 infection, but not disease severity. HCWs constitute a significant proportion of all COVID-19 lupus patients and represented 31% of all infections in our study. This is higher than the 4% to 29% range described in a meta-analysis, although severity and mortality in this group was low^35^. Data reflects the lack of adequate preparation and shortage of personal protective equipment at that time^35^. The other risk factor found was the presence of anti-SSA/Ro52 antibodies. Ro52/SSA, also known as TRIM21, is one of the major autoantigens recognized by the anti-Ro/SSA antibodies in lupus^36^. Patients with anti-SSA/Ro52 antibodies had lower TRIM21 expression compared with anti-SSA/Ro52 negative patients, and this reduction was more significant following COVID-19 infection. TRIM21, a cytosolic ubiquitin ligase, belongs to the tripartite motif-containing (TRIM) super family and has antiviral properties related to IFN signaling pathway regulation, the production of inflammatory mediators via NF-κB signaling, and by enhancing the Th1/Th17 response^37–40^. Knockdown of TRIM21 has been shown to impair innate immune response to viral infection^41^. Our in vitro studies showed that TRIM21 reduction decreased IL-4 and IFNγ cytokine expression levels. IL-4 and IFNγ induce cytotoxic T lymphocytes that provide an effective barrier to the establishment of viral infections^42,43^. By inhibiting TRIM21 and related cytokines, anti-SSA/Ro52 antibodies may impair the innate immune response in lupus patients contributing to a higher risk for COVID-19.

Severe disease occurred in 7 patients (24.13%), of whom 4 required ICU admissions and one died. Although limited by small numbers, these rates are higher compared to the ICU admission rates in the general population (13.79% vs 0.86%). Like previous studies^44,45^, our data did not show any association between COVID-19 severity and immunosuppressants or corticosteroids use. However, baseline hypocomplementemia, a classic feature of SLE and marker of disease activity, was a risk factor for severe COVID-19. The role of complement activation and its contribution to illness severity is being increasingly recognized^46–49^. The complement system is an arm of the innate immune response whose reactive cascade of cleavage products can coordinate the inflammatory antimicrobial response at sites of infection. However, complement activation is also associated with the excessive inflammatory response seen in patients with severe COVID-19, and the presence of a complement-mediated microvascular injury syndrome^50–53^.

Although the sample was small, no increased lupus activity in COVID-19 patients was observed. This may be due to better compliance with HCQ in view of early reports of its efficacy in COVID-19 prevention, the increment of corticosteroids received in severe cases and the Th2 skew observed during convalescence. Overall, whether SARS-CoV-2 can trigger SLE flares is still controversial^54–56^. For those studies showing a de novo SLE diagnosis or worsening disease following COVID19, caution needs to be taken since certain SLE clinical diagnostic criteria overlap with COVID-19 symptoms and autoantibodies can occur transiently in response to infections. In addition, it is difficult to evaluate SARS-CoV 2-related worsening SLE disease in a setting where patients suffered from psychological distress, faced difficulty in healthcare access and withdrew medication due to financial constraints or fear of being immunocompromised^57^.

SARS-CoV-2 IgG antibody prevalence was 8.33%. Our findings differ from the 16% reported by Saxena A et al.^45^. Cohorts were similar except by the higher rates of Hispanic ethnicity in their study having a higher antibody production. Reported seroconversion rates after SARS-CoV-2 symptomatic infection range from 91 to 99% in the general population^58^. SARS-CoV-2 IgG antibodies are generally detected 2 weeks after infection and have a variable durability. As previously reported, most lupus patients (75%) with RT-PCR-confirmed SARS-CoV-2 infection developed SARS-CoV-2 IgG antibodies despite 69% being on immunosuppressants, and titers were correlated with disease severity^59–61^. Lack of antibody response was associated with prednisone doses ≥ 7.5 mg/day. The impact of corticosteroids on the humoral response is unclear. Whereas short courses have been associated with decrease serum of IgG and IgA concentrations, studies using pulses has not shown a detrimental effect^62,63^. We also found seronegative patients to receive more mycophenolate mofetil. MMF is known to suppress the humoral immune response to influenza vaccine in kidney transplant^64^ and to mRNA COVID-19 vaccines in solid organ transplant recipients^65^. Although seroprevalence in healthy individuals during the same period^66,67^ ranged from 4 to 20%, in our cohort it was much lower (0.78%). In the questionnaire, most patients reported that they implemented strict protective behaviors to avoid exposure.

Overall, the serological response was sustained, but antibody loss over serial measurements was observed in patients by week 24. They were patients with mild COVID-19 and receiving immunosuppression. Previous studies reported that SARS-CoV-2 specific antibodies disappeared in convalescent COVID-19 patients within 3 to 6 months, mainly in mild disease^68–70^. However, we did not find any significant immunological profile.

SLE is characterized by an aberrant immune response and many immunopathogenic mechanisms overlap with those described in COVID-19. Due to restrictions in obtaining samples during the acute infection, we characterized only the cross-sectional and longitudinal SARS-CoV-2 cellular response in convalescent patients. While studies in the general population showed variable CD8^+^ T cell responses, we found a significant reduction of CD8^+^ T cell frequency compared to non-infected lupus patients that progressively recovered, and no differences in CD4^+^ T and NK cells^71^. Unlike the reported predominant Th1 response with undetectable antigen-specific Th2 or Th17 responses in general convalescent COVID-19 patients, we found a predominant Th17 with a mild Th2 response, more pronounced in severe disease and especially in those with pneumonia. Data on T-cell subtype changes is limited, with few longitudinal studies or studies evaluating patients with severe disease^72^. Th2 and Th17 responses have been found to be detrimental for recovery in severe COVID-19^72^, in part due to their association with lung injury^73^. The increase in Th17/Treg ratio may contribute to the uncontrolled release of pro-inflammatory cytokines and chemokines, which could result in aggravated inflammatory responses. While elevated Th17 cells have been detected in SLE^74^, in the absence of other observed lupus activity, this increment appears to be more strongly correlated to COVID-19 disease severity and poor outcome. Most patients with a persistent Th17 response developed lung fibrosis and thrombotic events during follow-up. A recent study has suggested a possible role of Th17 as a predictor of progression to critical disease in COVID-19 hospitalized patients^75^. Consequently the predominant Th17 response observed in our SLE patients could be related more for SARS-CoV-2 infection rather than SLE activity.

Several reports have demonstrated that SARS-CoV-2 elicits a protective immune memory response after recovery^76,77^. We report B and T-cell responses in PBMCs from lupus patients 6 months post-infection despite immunosuppressive therapy. Overall, the development of B-cell memory to SARS-CoV-2 was significant and sustained. Notably, it has been reported that memory B cells targeting the Spike protein or RBD have been detected in most COVID-19 cases^78,79^. SARS-CoV-2-specific CD4^+^ and CD8^+^ T cells have been detected in 100% and ~ 70% of convalescent individuals a short time after resolution^79^. In severe COVID-19 cases, it has been reported that CD8 + T cells exhibit a high proportion of CD57 + terminal effector cells, together with significant decrease of naïve cell population leaving unresolved inflammation 6 months after infection^80^. Likewise, we found decreased CD8^+^ naïve T cells, increased TEMRA and CD8^+^ memory T cells during the study period regardless of COVID-19 severity.

There are several limitations in our study. First, there are no data during COVID-19 infection or the first weeks after discharge for comparison. Second, the relatively small number of patients evaluated in some of the sub-studies did not allow us to draw strong conclusions. Further larger studies are needed to validate the findings reported here. Third, although antibody testing was mostly done as standard of care, there may be some degree of bias regarding which patients were willing to travel to the hospital. SLE patient could have strict protective behaviors to avoid COVID-19 infection exposure and it could also create a bias of the results. Fourth, we were not able to perform neutralizing antibodies assay due to laboratory biosafety limitations. Fifth, we did no measured antigen specificity for memory cells. Finally, some of the immune modifications may have been influenced by the treatment received in severe COVID-19 such as steroids and anti-IL6.

## Conclusions

In summary, this study shows that SLE patients are at higher risk of SARS-CoV-2 infection compared to the reference population. We identified HCWs and anti-SSA/Ro52 antibody positivity as risk factors for infection and hypocomplementemia for disease severity. Our study also extends an understanding of the humoral and cellular immune response of SLE patients to COVID-19. Most patients developed a sustained humoral and memory immune response despite immunosuppressive therapy. Convalescent lupus patients had a predominant Th17 response in severe cases with poor outcome.

## Supplementary Information

Supplementary Information.

## Data Availability

All data relevant to the study are included in the article or uploaded as supplementary information.
