# Supplementary material for: Humoral and cellular response in convalescent COVID-19 lupus patients
Source: Sci Rep. 2022 Aug 12;12:13787. doi: 10.1038/s41598-022-17334-5 (PMC9374301; doi:10.1038/s41598-022-17334-5)
Supplement: Supplementary file 1 — Supplementary Information. [file 41598_2022_17334_MOESM1_ESM.pdf]

Supporting Information

Humoral and cellular response in convalescent COVID-19 lupus patients

Cristina Solé[1], Sandra Domingo[1], Xavier Vidal[2] , Josefina Cortés-Hernández[1]

[1]Department of Rheumatology, Lupus Unit, Hospital Universitari Vall d´Hebron, Institut de Recerca (VHIR), Universitat Autònoma de Barcelona, Barcelona, Spain. [2] Clinical Pharmacology Service, Department of Pharmacology, Therapeutics and Toxicology, Fundació Institut Català de Farmacologia, Hospital Universitari Vall d'Hebron, Universitat Autònoma de Barcelona, 08035 Barcelona, Spain

INDEX

1. **SI Materials and Methods**
2. **References**
3. **Supplementary Questionnaire**
4. **Supplementary Figures**
5. **Supplementary Tables**

1. SI Materials and Methods

Patients' clinical characteristics and samples

COVID-19 disease was classified according to severity. Mild disease was defined by symptomatic cases with or without pneumonia not requiring intensive care. Severe disease was defined by the requirement of critical care with ≥1 of these criteria: dyspnoea and respiratory rate ≥30/min, blood oxygen saturation ≤93%, PaO2/FiO2ratio <300 mmHg, and lung infiltrates on CT scan >50% within 24–48 h, or those who exhibited respiratory failure, septic shock, and/or multiple organ dysfunction/failure (1).

All COVID-19 patients admitted into hospital received lopinavir/ritonavir and azithromycin. Seven (24.14%) had their corticosteroid dose increased and 4 received i.v tocilizumab. Immunosuppressant therapy was interrupted or decreased during the admission period.

Biological samples

Peripheral blood mononuclear cells (PBMCs) from convalescent or unexposed lupus patients were isolated from whole blood by Ficoll-Hypaque gradient centrifugation (Vacutainer CPT, BD Biosciences). Tubes were centrifuged at 3000 rpm for 30 minutes at room temperature. After that, the section containing peripheral blood mononuclear cells was clearly visible and collected using a pipette. Serum samples were obtained after 15 minutes of centrifugation at 2000 rpm for antibody detection.

COVID-19/SARS-CoV-2 clinical samples were inactivated using validated protocols according to specimen type under the biosafety level 2 laboratory guidelines (2). Thus, samples for flow cytometry were fixed adding 4% formaldehyde for 20 minutes at room temperature, samples for RNA extraction were chemically inactivated using AVL buffer (Qiagen, Hilden, Germany) and samples for antibody detection were heat-inactivated (30 minutes at 56ºC).

Detection of SARS-CoV-2 antibodies

Three serological test were performed in order to determinate IgG and IgM antibodies for SARS-CoV-2. Two of them were qualitative and one quantitative.

- ***COVID-19 IgG/IgM Rapid Test Kit***

The point-of-care test (Elabscience, UNCOV-4 COVID-19 IgG/IgM Rapid Test Cassette) is a lateral-flow immunochromatographic assay for qualitative differentiation between IgG and IgM against the receptor binding domain of SARS-CoV-2 spike (S) protein, which yields results in 10 minutes. The manufacturer reported sensitivity of 97,2% for IgG and 87,9% for IgM and specificity of 100% for both IgG and IgM, using reverese transcription polymerase chain reaction (RT-PCR) as the gold standard. A verification study (3), done by the National Centre for Microbiology as preparation for ENE-COVID, returned a sensitivity of 82,1% for IgG and 69,6% for IgM in fingerprick blood samples and a specificity of 100% for IgG and 99,0% for IgM. Due to the lower sensitivity and specificity of IgM, its shorter duration, and the heterogeneity of results observed in initial IgM readings, results for the point-of-care test reported here are based only on IgG.

- ***CLIA assay***

The Maglumi™2019-n-Cov IgG and IgM are fully automated quantitative chemiluminescent immunoassays (CLIA) using magnetic microbeads coated with SARS-CoV-2 recombinant antigen labelled with ABEI, a non-enzyme small molecule with a special molecular formula that enhances stability in acid and alkaline solutions. The IgM and IgG assays were performed on serum samples, according to the manufacturer's instructions, on the Maglumi™ 800 analyser (Snibe Diagnostic, Shenzhen, China). The thresholds of positivity for these automated immunoassays are 1.0 AU/mL for IgM and IgG.

- ***ELISA assay for antibody titration***

Patients positive for rapid test kit or CLIA assay were confirmed, and titres measured using an in-house ELISA assay. We coated 96 well-microtiter ELISA plates with 50 µl of 2µg/mL recombinant SARS-CoV-2 RBD (receptor binding domain) proteins (Genetex). After overnight incubation at 4ºC, plates were washed three times with phosphate-buffered saline (PBS) containing 0.1% vol/vol Tween-20 (PBST) and blocked with blocking solution (PBS containing 2% wt/vol non-fat dry milk and 1% BSA) for 1 hour at room temperature. After three washes with PBST, the serum samples were serial diluted in PBS (1/200) and added to the appropriate wells and incubated for 2 hour at room temperature. Wells were washed with PBS and 100 µL of horseradish peroxidase–conjugated goat anti–human IgG (for IgG antibody titter detection) and IgM (for IgM antibody titter detection) antibodies solution (1/1000) were added to each plate, respectively, and incubated at 2 hours. 100 µL of tetramethylbenzidine substrate (Sigma-Aldrich) was added for 25 minutes at 37ºC in the dark. The reaction was stopped with a 2M H_2SO_4 solution. Optical density (OD) was measured at 450 nm. All assays were run in triplicate.

Flow cytometry analysis (FACS)

For flow cytometry analysis, we used two cohorts of PBMCs samples. First, we performed a cross-sectional analysis in PBMCs samples from COVID-19 convalescent patient at 12 weeks after the onset of symptoms to be compared with unexposed lupus patients (N=20 in each group). Second, we performed a longitudinal analysis with patients of the cross-sectional study using PBMCs samples from COVID-19 convalescent patients at 6, 12 and 24 weeks after onset of symptons (n=12 at each time point). PBMCs for flow cytometry were fixed adding 4% formaldehyde for 20 minutes at room temperature. During the fixation step, SARS-CoV-2 was inactivated according to the protocol. Thereafter, conjugated monoclonal antibodies were added for cell surface staining during 15 minutes at room temperature. Cell phenotype was analysed by seven-color flow cytometry (LSR Fortessa, BD Biosciences, Erembodegem, Belgium). Data were analysed using FCS Express 4 Flow Research software (all BD Biosciences, Erembodegem, Belgium).

RNA extraction and RT-qPCR

PBMCs from COVID-19 patients were chemically inactivated using AVL buffer (Qiagen, Hilden, Germany) for RNA extraction. Then, RNA were obtained using RNeasy Mini Kit (Qiagen, Hilden, Germany) following manufacturer's instructions. Total RNA from cultured cells was also extracted after the cell lysis with RNeasy Mini Kit (Qiagen). The yield and the quality of RNA from cell cultures were assessed by measuring its absorbance at 260nm and 280nm with Nanodrop. Ratios of A260/A280 between 1.8 and 2.1 were considered acceptable to use the RNA for the subsequent experiments.

Once RNA was obtained, 1ug of total RNA was reverse transcribed into cDNA using the High-Capacity cDNA Reverse Transcription Kit (Applied Biosystems) with the thermal cycler program: 25ºC for 10min, 37ºC for 120 min and 85ºC for 5min. Gene expression was assessed by TaqMan gene expression assays gene expression assays (FAM dye-labeled MGB probe, Applied Biosystems). Using 384 well plates in the ABI PRISM 7900 thermocyclers respectively at 50ºC for 2 min, 95ºC for 10min, followed by 40 cycles of 95ºC for 15s and 60ºC for 1 min. Obtained data was normalized based on the expression the endogenous control gene GAPDH (Hs02786624_g1, Thermofisher scientific, Waltham, MA, USA) and TaqMan gene expression assay are cited on Table S2.

Immunofluorescence studies

PBMCs from unexposed COVID-19 SLE patients with or without anti-SSA/Ro52 antibodies were seeded in sterile glass coverslips in 24 well-plates and incubated overnight at 37ºC for adherence. Cells were cultured with RPMI media (RPMI, 10% FBS, 1% Pen/Strep, 2 mM/L-Glutamine) (Gibco, Life Technologies, Carlsbad, CA, USA)) and stimulated with RBD, S1 spike or sterile PBS for 24 hours (100ng/well, Bioscience, Cambridge, UK). After that, cells were washed with PBS and then fixed for 15 minutes in 4% PFA followed by permeabilization with 0.1% TritonX-100 for 10 minutes. Blocking solution (BSA 5%) was added for 1 hour at RT and primary Human anti-TRIM21 (1:250, Cloud-Clone Corp, Katy, TX, USA) was incubated at 4ºC overnight being followed by a secondary Alexa-488-conjugated anti-rabbit IgG (1:500, Abcam, Cambridge, UK) at room temperature for 2h. DAPI was used to visualize the nucleus. Images were captured using Olympus BX61 microscope and percentage of positive cells were obtained using ImageJ program.

2. References

1. Rajesh TG, Lynch JB, Del Rio C. Mild or Moderate Covid-19. New Engl J Med. 2020; 383:1757-1766.

2. Vanderbilt Institutional Biosafety Committees Policy. BSL-2 with Enhanced Containment Practices for Activities Involving Potential COVID-19/SARS-CoV-2 Specimens, as of 28 Abril 2020. URL: https://www.vumc.org/safety/sites/default/files/public_files/bio/BSL-2-with-Enhanced-Containment-%28COVID-19-clinical-samples%29-4.14.2020.pdf. Accessed 27th Abril 2020.

3. Pollán M, Pérez-Gómez B, Pastor-Barriuso R, Oteo J, Hernán MA, Pérez-Olmeda M, et al. Prevalence of SARS-CoV-2 in Spain (ENE-COVID): a nationwide, population-based seroepidemiological study. Lancet. 2020; 396:535-544.

3. Supplementary Questionnaire

This is a short survey to gather information on how patients with Systemic Lupus Erythematosus have experienced the global COVID-19 pandemic. The Lupus Unit (Rheumatology Research Group, VHIR – Vall d'Hebron Research Institute), assures that all the information provided will be confidential and the collected data will be used in an anonymous form exclusively for this biomedical research study, according to local data protection law (LOPD-GDD 3/2018) and EU 2016/679 regulation article 13 (GDPR). Applicable Good Clinical Practice (GCP) guidelines will be followed. Within this interview, patients will be identified with a unique numeric ID, in order to ensure anonymity and traceability. All the data will be stored in an electronic password-protected database that is only accessible to authorized personnel.

Do you give consent for this interview?

- ☐ *Yes.*
- ☐ *No (interview was stopped).*

- **Name and Surname:**
- **Telephone number:**
- **Email:**
- **Age:**
- **Sex:**
- **Diagnosis:** SLE / CLE
- **City and neighborhood where did you reside during COVID-19**

- **Do you have any of these other following conditions?**
  - ☐ *Hypertension*
  - ☐ *Diabetes.*
  - ☐ *Chronic Lung disease: asthma, bronchitis…*
  - ☐ *Heart disease*
  - ☐ *Obesity*
  - ☐ *Renal disease*
  - ☐ *Depression*
  - ☐ *Others:*

- **Do you smoke?**
  - ☐ *Yes*
  - ☐ *No*
  - ☐ *If yes, how many cigarettes do you smoke? How many years have you been smoking?*

- **Do you drink?**
  - ☐ *Yes*
  - ☐ *No*
  - ☐ *How much?*

- **What is your occupation?**
  - ☐ *I am a student*

- ◻ *I work*
- ◻ *Others:*

If you work, what is your profession?

- **Did you have any of these symptoms the previous month? Select all the options you require.**

- ◻ *Fever less than 38ºC*
- ◻ *Fever over 38ºC*
- ◻ *Diarrhea*
- ◻ *Loss of smell or taste*
- ◻ *Pain when swallowing*
- ◻ *Cough*
- ◻ *Dyspnea or drowning symptoms*
- ◻ *Muscle discomfort or pain*
- ◻ *Headache*
- ◻ *Chest pain*
- ◻ *Weight loss*
- ◻ *Fever in the second week*

- **Have you been diagnosed with COVID-19?**

- ◻ *Yes, with positive PCR test*
- ◻ *Yes, possible SARS-Cov-2 infection due to compatible symptoms. No PCR test performed.*
- ◻ *No*

- **Have you had a serological test to see if you are immune to COVID-19?**

- ◻ *Yes*
- ◻ *No*

- **If you had COVID-19, have you been hospitalized?**

- ◻ *Yes, I was hospitalized in Intermediate Care.*
- ◻ *Yes, I was hospitalized in the Intensive Care Unit (ICU)*
- ◻ *Yes, I was hospitalized, I was in the ICU, and I was intubated.*
- ◻ *No, I stayed at home.*
- ◻ *If hospitalized, at what hospital?*

- **If you had COVID-19, how many days did the symptoms last?**

- ◻ *Less than a week.*
- ◻ *Between one and two weeks*
- ◻ *More than two weeks*

- **If you were admitted into hospital by COVID-19, how many days were you there?**

- ◻ *Less than a week*
- ◻ *Between 7-14 days*
- ◻ *More than 14 days*

- **When you were admitted, what happened to your chronic lupus treatment?**

- ◻ *It was removed*

- ◻ *It was increased*
- ◻ *It was not changed*

- **After COVID-19, do you have any sequelae left?**

  - ◻ *No, I'm perfect.*
  - ◻ *Yes, I have slight trouble breathing and slight fatigue.*
  - ◻ *Yes, I have important sequelae and doctors will continue to do checks on me.*

- **What was the reason for the infection with COVID-19?**

  - ◻ *Close contact of a family member, neighbor, friend, or person close to you.*
  - ◻ *Attended a crowded event (concert, theater, demonstrations, etc.).*
  - ◻ *Taking public transport.*
  - ◻ *Traveled to another country.*
  - ◻ *For the work I have.*
  - ◻ *I don't know.*
  - ◻ *None of the above.*

- **Do you have any relatives or close friends who have been diagnosed with COVID-19? If so, how many people?**

- **Do you take measures to avoid infection?**

  - ◻ *Yes*
  - ◻ *No*
  - ◻ *I don't know*

- **Do you think you have complied with confinement restrictions?**

  - ◻ *Yes*
  - ◻ *No*

  If so, how many times have you been out on the street a week? Can you specify the reason?

- **If yes, what measures do you take to prevent infection? Select the ones that apply**

  - ◻ *I did not go out at all*
  - ◻ *If I had to go out, I went hours that not many people were around and with FFP2 masks*
  - ◻ *If I had to go out, I kept the distance required*
  - ◻ *I sent my relatives to do my shopping*
  - ◻ *I shop my groceries on-line.*
  - ◻ *I wore a mask*
  - ◻ *I avoid people visiting me even if they were close relatives*
  - ◻ *I wash my hands with gel often*
  - ◻ *If my husband works, I kept myself almost isolated in my room*
  - ◻ *Others*

- **How did you feel during the confinement? You can select more than one option.**

  - ◻ *Stressed, sad or anxious*
  - ◻ *Muscle aches*
  - ◻ *Joint pain*
  - ◻ *Fever*
  - ◻ *New skin lesions*
  - ◻ *Chest pains*
  - ◻ *Worried about the situation but I got along well*

- **Could you say that during the confinement did you have a flare of your lupus disease?**

  - ◻ *Yes*
  - ◻ *No*
  - ◻ *I'm not entirely sure*

- **Which symptoms related to lupus did you have over this period? Select all required**

  - ◻ *None*
  - ◻ *Skin rash*
  - ◻ *Joint pain and swelling*
  - ◻ *Hair fall*
  - ◻ *Mouth ulcers*
  - ◻ *Pleuritis or pericarditis*
  - ◻ *Fever*
  - ◻ *Others: specify*

- **If you need to consult something about Lupus with your specialist, have you been able to do so during the COVID-19 confinement?**

  - ◻ *Yes, specialist was available, and I had no problems.*
  - ◻ *Yes, but it has not been easy.*
  - ◻ *No, it has been impossible.*

- **Did you have a visit with your specialist during the COVID-19 situation?**

  - ◻ *Yes, but it was a telephone visit.*
  - ◻ *Yes, and it was face-to-face.*
  - ◻ *Yes, but I could not attend, and the visit was rescheduled.*
  - ◻ *No.*

- **Do you think that information on the situation of COVID-19 specific for patients with Lupus has been provided? You can choose from several options.**

  - ◻ *Yes, I obtained it from television.*
  - ◻ *Yes, I obtained from the social network of the Catalan Lupus Foundation.*
  - ◻ *No, I felt uninformed.*

- **For your Lupus treatment, do you take hydroxychloroquine (Dolquine) or chloroquine (Resochin)?**

- ☐ *Yes, dolquine*
- ☐ *Yes, resochin*
- ☐ *None*

- **If so, did you have problems to obtain it in pharmacies?**

  - ☐ *Yes. But I was finally able to get it at the pharmacy*
  - ☐ *Yes, and I had to go to my referral Hospital's pharmacy to find it.*
  - ☐ *No*

- **What medication are you currently taking for your Lupus treatment?**

- **During the COVID-19 pandemic, did you have any changes in your chronic Lupus treatment?**

  - ☐ *Yes, I have had my corticosteroids removed (Dacortin, Urbason, Fortecortin, etc.)*
  - ☐ *Yes, I have had my immunosuppressive treatment withdrawn (Myfortic, methotrexate, Imurel, etc.)*
  - ☐ *Yes, I have had my biological treatment withdrawn (Belimumab, Enbrel, Rituximab, etc.)*
  - ☐ *Yes, I have had both corticosteroids and immunosuppressive or biological therapy removed*
  - ☐ *Yes, my corticosteroids have increased*
  - ☐ *Yes, I have increased my dose of immunosuppression or biologic therapy.*
  - ☐ *No, it has continued all the same.*

- **Have you been diagnosed of any other medical condition?**

  - ☐ *Yes*
  - ☐ *No*

  If so, which one and which treatment have been prescribed

4. Supplementary Figures

Figure S1. Serum hydroxychloroquine levels in patients with antimalarial treatment. a) A calibration curve (2–2,000 ng/ml) was generated to validate the method (r=0.999). **b)** Levels of hydroxychloroquine in serum were similar between lupus COVID-19 infected and non-infected patients.

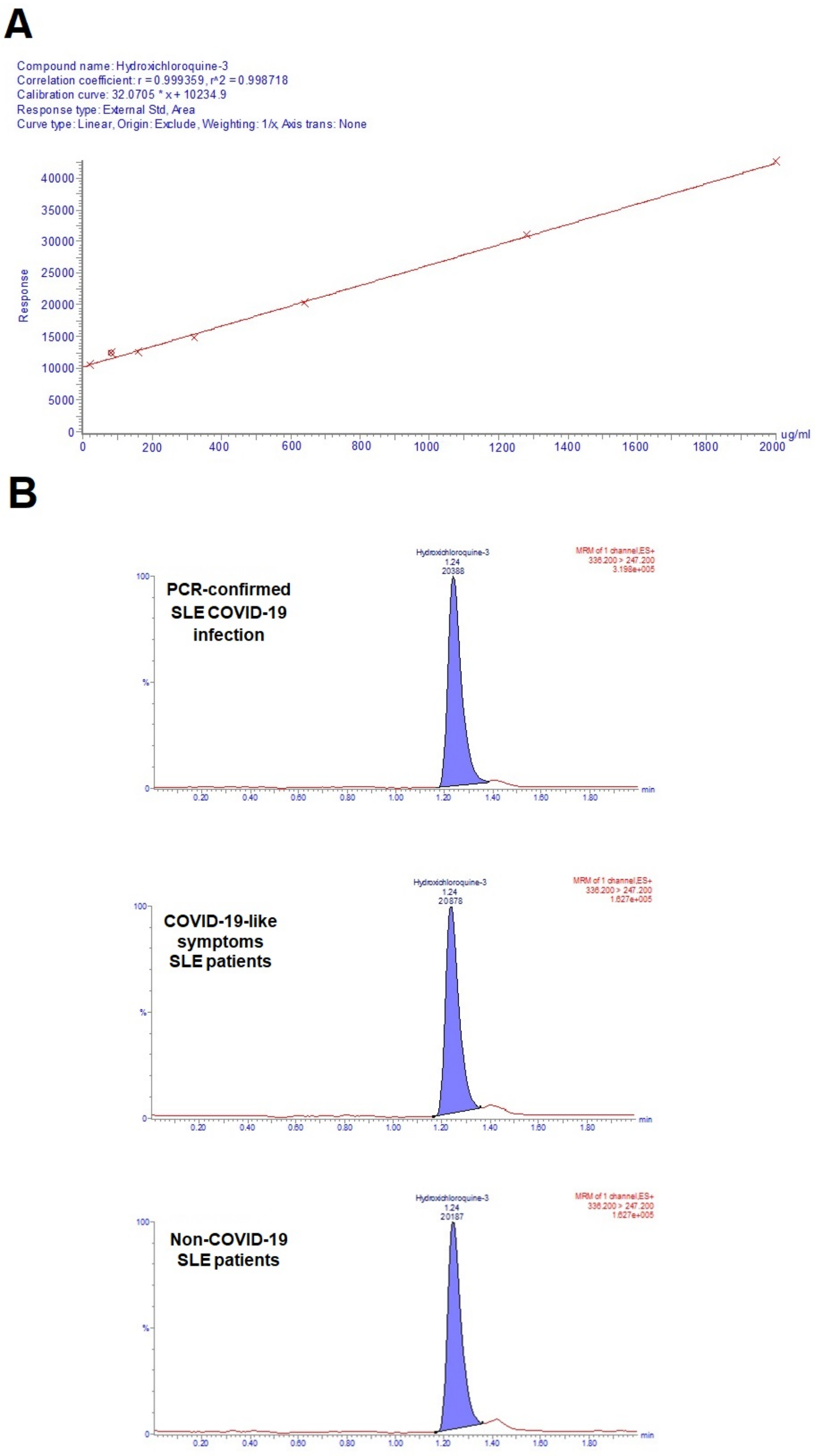

Figure S2. Correlation between TRIM21 expression levels and anti-SSA/Ro52 titers. A significant correlation was founded between TRIM21 expression levels and anti-SSA/Ro52 values in RT-PCR confirmed COVID-19 lupus patients with anti-SSA/Ro52 positive antibodies (p=0.0251, N=13).

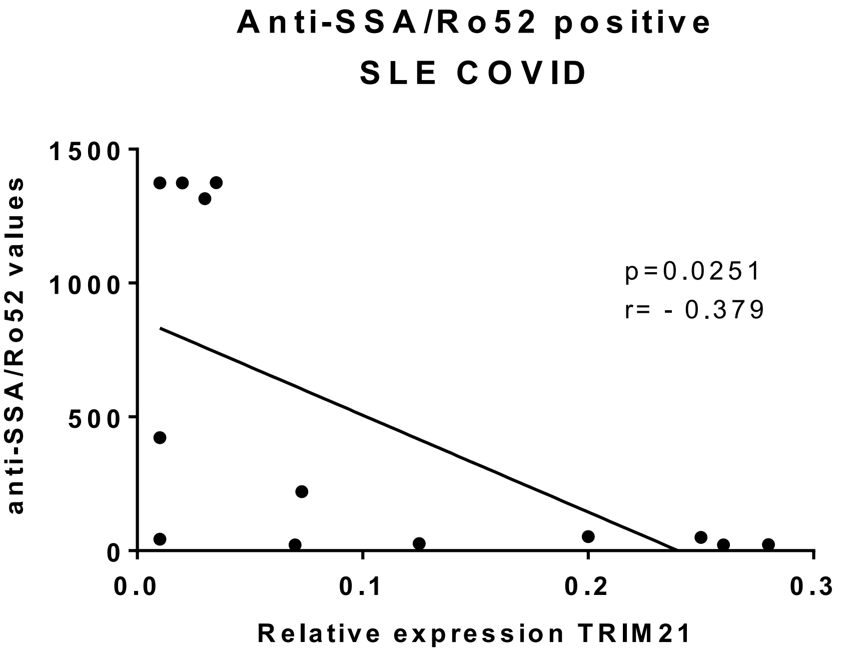

Figure S3. PBMCs from unexposed SARS-CoV-2 lupus patients with anti-SSA/Ro52 positive or negative antibodies were stimulated with RBD or S1 protein to analyze their gene expression levels. No significant differences were founded between anti-SSA/Ro52 positive or negative patients in *INFA1*, *IRF4* or *IL17A* expression levels.

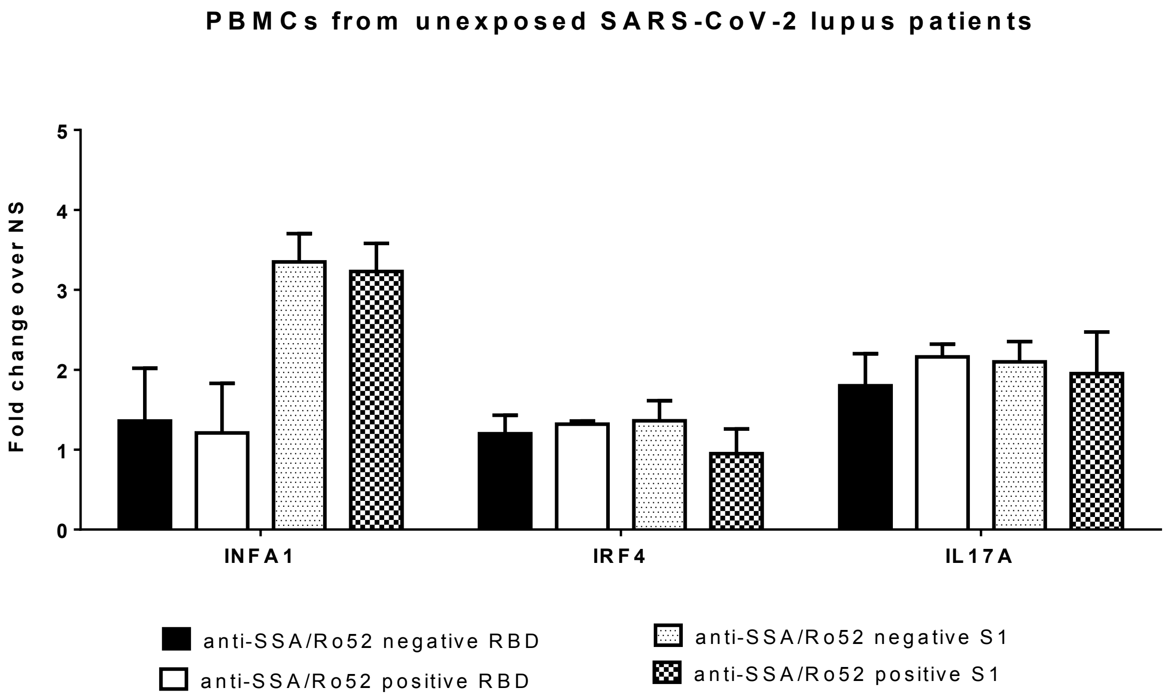

Figure S4. Flow cytometry analysis of peripheral blood mononuclear cells (PBMCs) from COVID-19 convalescent lupus patients at week 12 after onset of symptoms and unexposed lupus patients. A) Representative gating of CD19$^+$CD27$^-$IgD$^+$ and CD19$^+$CD27$^+$IgD$^-$ for the quantification of naïve and memory B cells from lupus patients. **B)** Gating of live CD38$^+$CD27$^+$ plasmablats / plasma cells (PBs/PCs) **C)** Representative gating of CD3$^+$CD8$^+$ T cells.

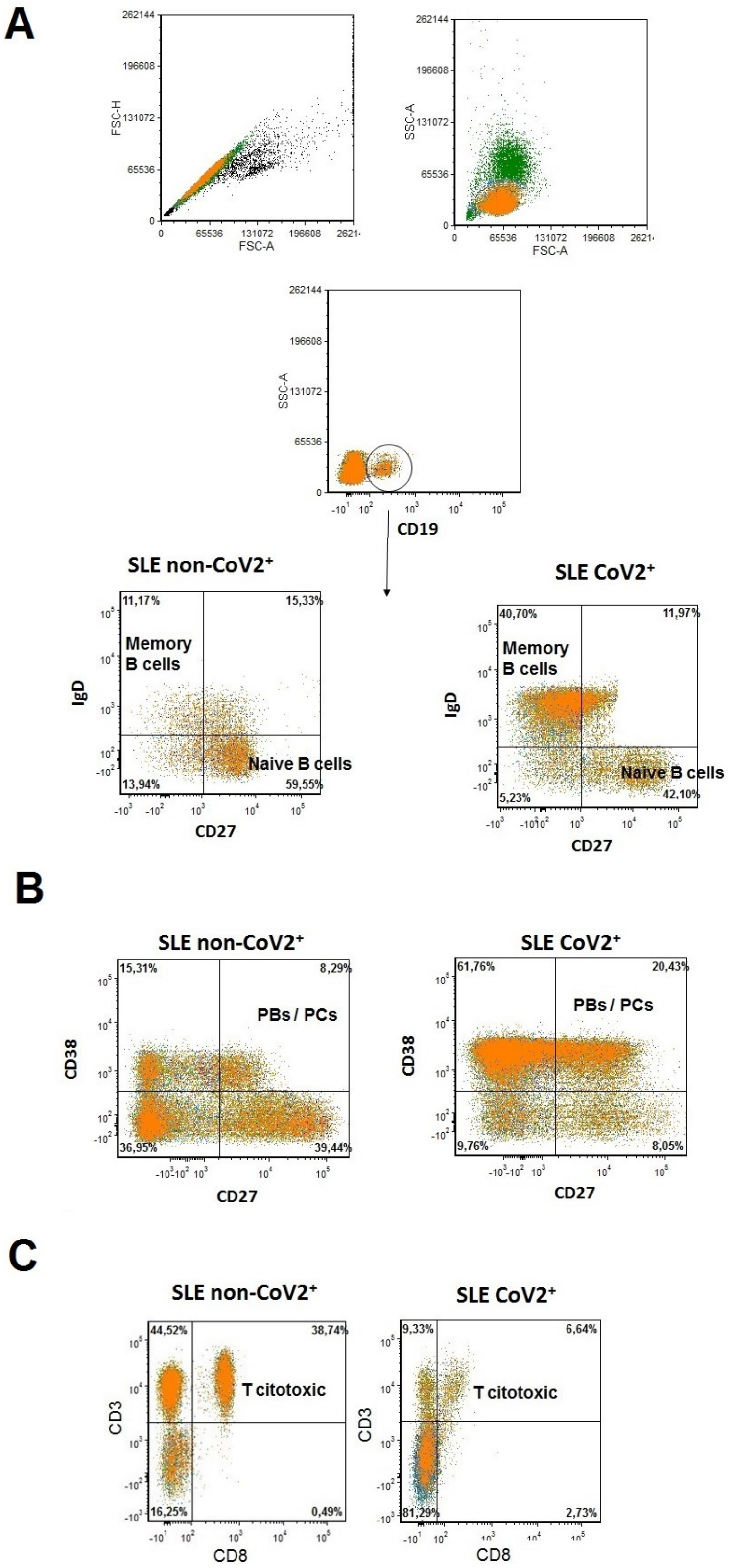

Figure S5. Percentage cells obtained by flow cytometry in convalescent COVID-19 lupus patients according to disease severity (N=20). An increase of CD8$^+$ T memory was observed in severe disease group (p=0.0279, 36.22% vs 24.60%).

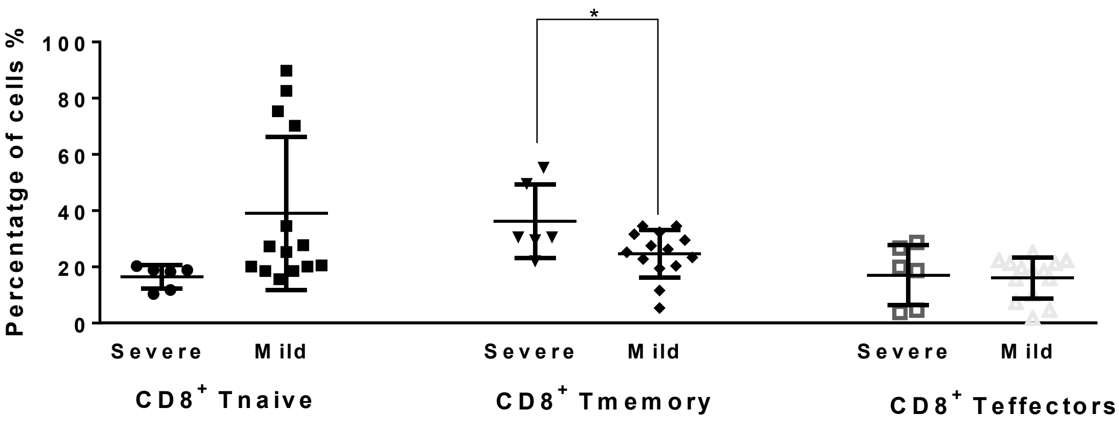

Figure S6. Percentage cells obtained by flow cytometry between SARS-CoV-2 infected and non-infected lupus patients at week 12. No differences were observed in CD4+ T cells, CD69+ T cells **(A),** NK cells **(B)** or subsets of CD4+ T cells **(C).**

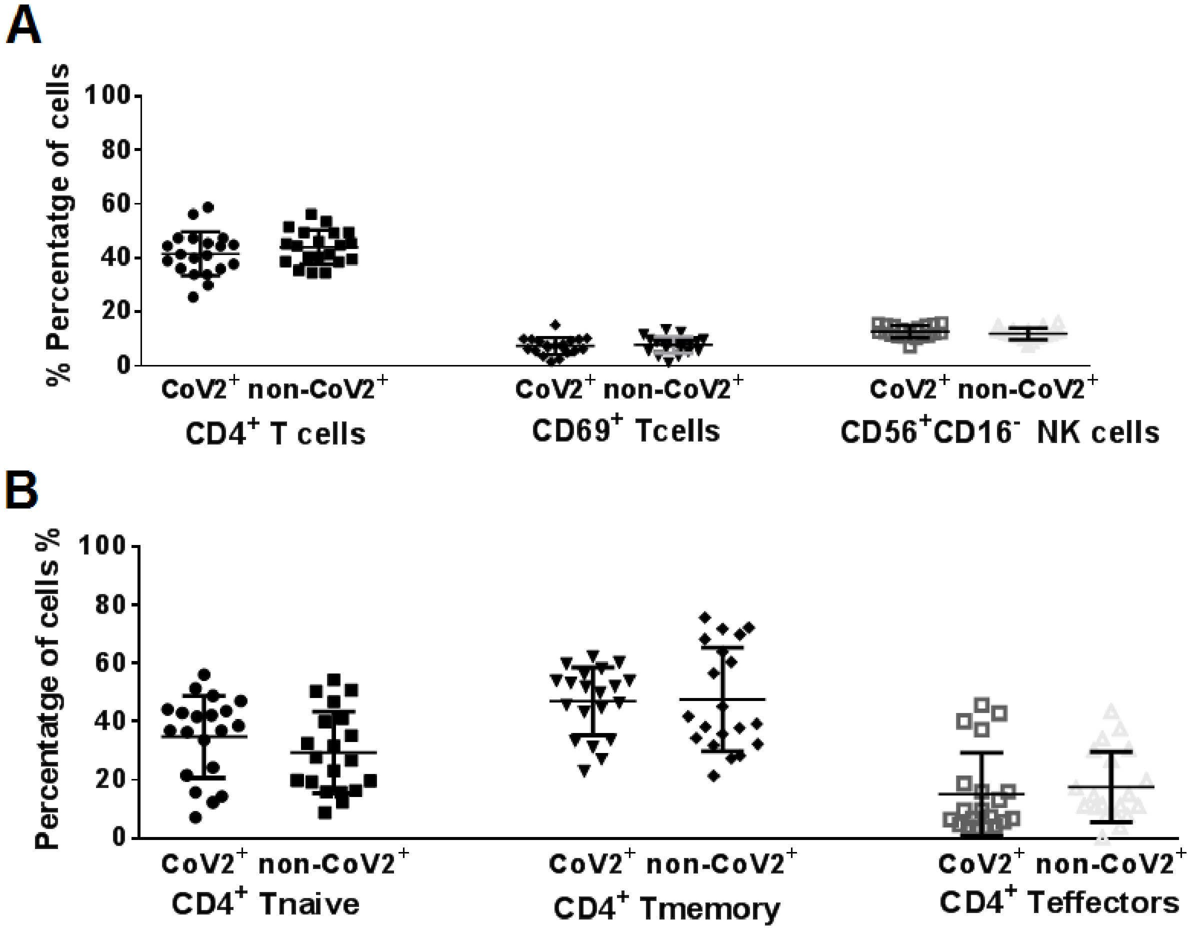

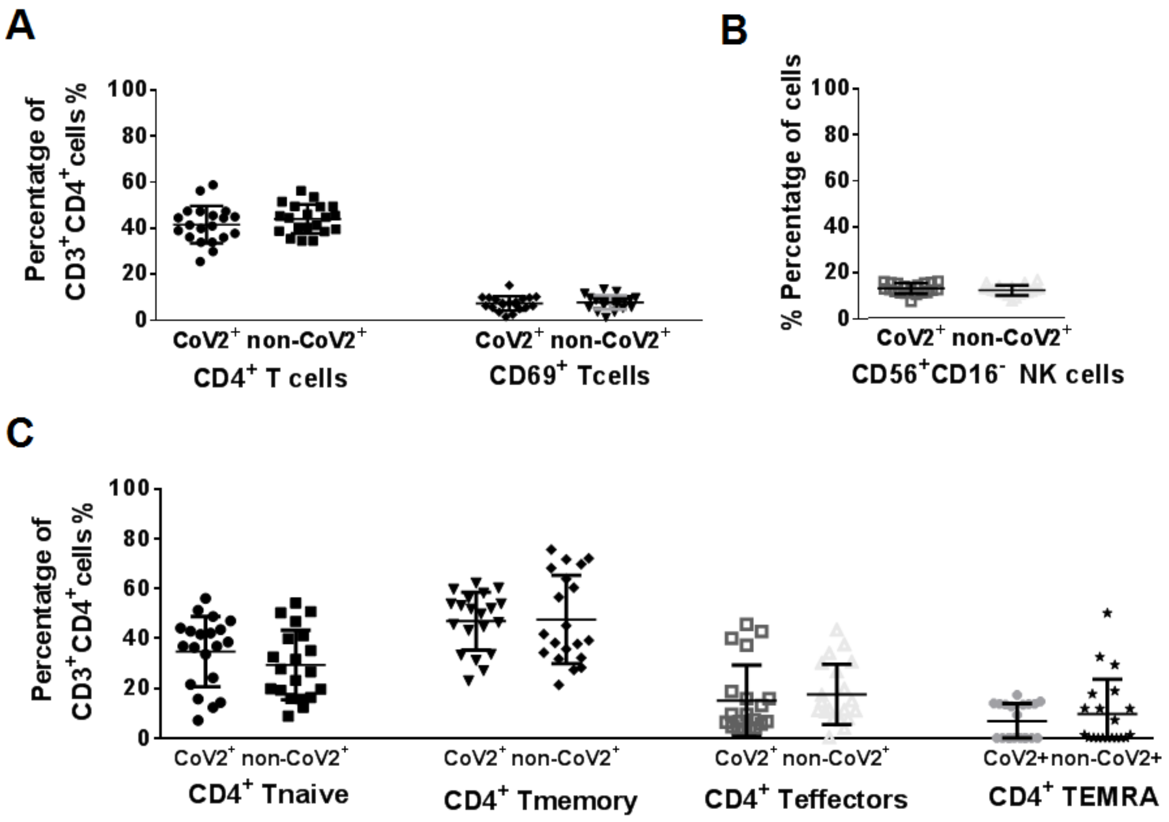
A
B
C
Percentatge of CD3+CD4+ cells %
100
80
60
40
20
0
CoV2+ non-CoV2+
CD4+ T cells
CoV2+ non-CoV2+
CD69+ Tcells
% Percentatge of cells
CD56+CD16- NK cells
CD4+ Tnaive
CD4+ Tmemory
CD4+ Teffectors
CoV2+non-CoV2+
CD4+ TEMRA

Figure S7. Type I gene interferon signature in PBMCs from COVID-19 SLE patients at week 12 after onset of symptoms and from unexposed lupus patients. RNA expression analysis was performed in RNA extracted from PBMCs and no significant differences were observed between patients´ cohorts (N=20 in each).

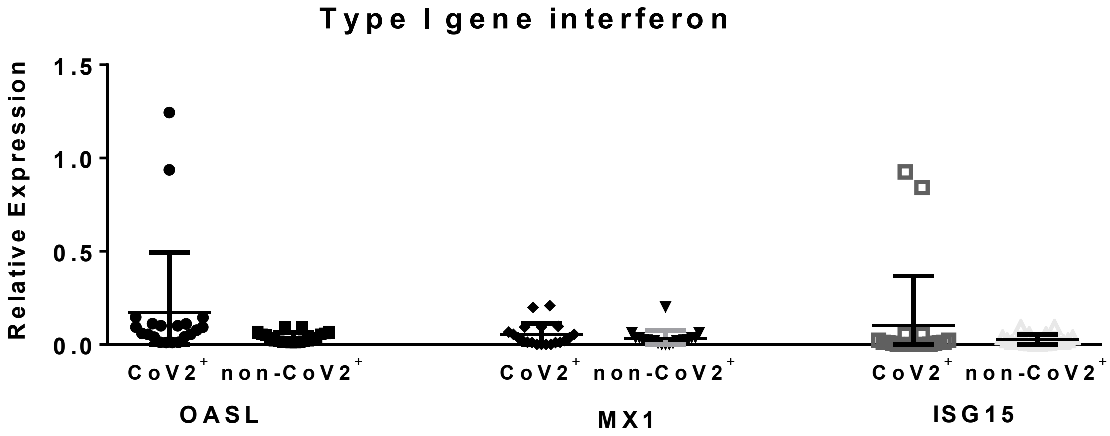

Figure S8. Flow cytometry longitudinal B cell analysis in convalescent COVID-19 lupus patients according to disease severity (N=12). No differences in B cells (naïve, memory o plasma cells / plasmablats) were observed between mild and severe COVID-19 lupus patients at week 6, 12 and 24 weeks after onset of symptoms.

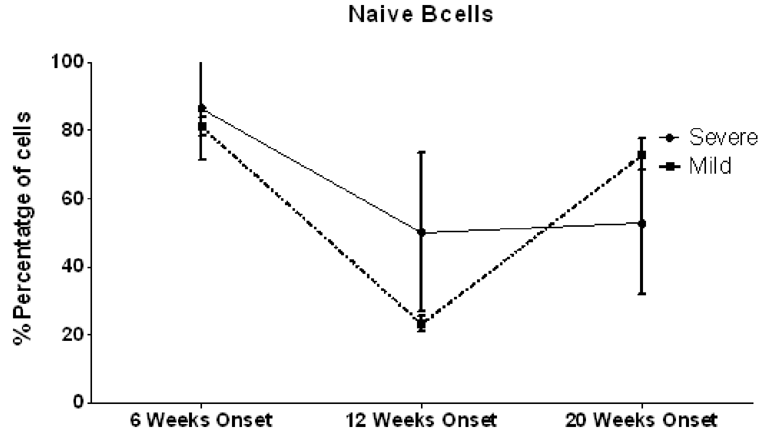

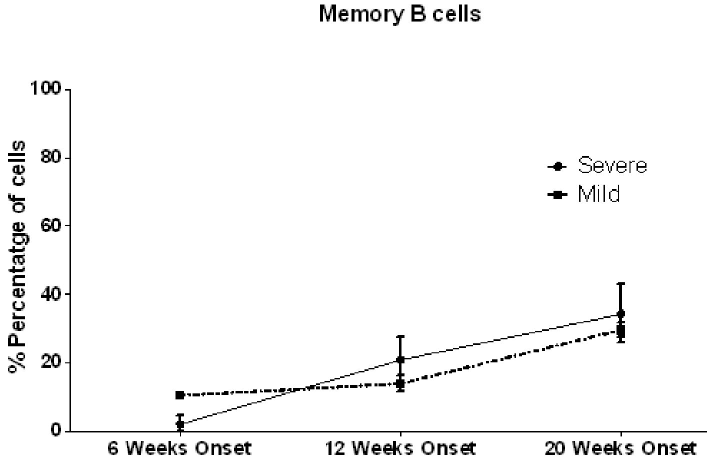

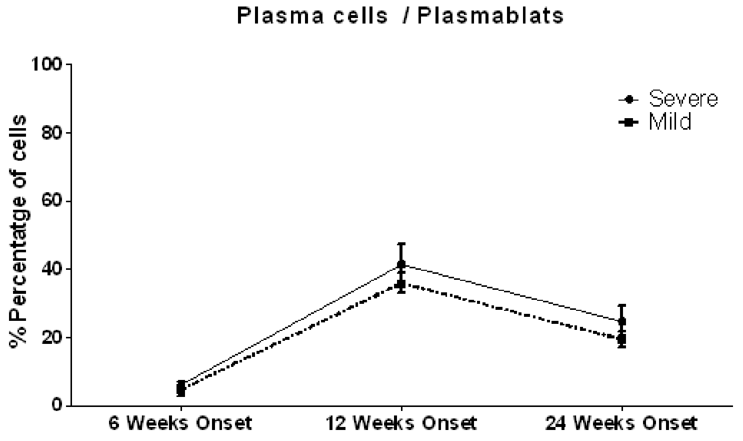

Figure S9. Flow cytometry longitudinal CD8+ T cell analysis in convalescent COVID-19 lupus patients according to disease severity (N=12). Differences between severe and mild were found only at 6 and 12 weeks after onset of symptoms in T naïve (p=0.002 and 0.017, respectively) and T effector (p=0.005).

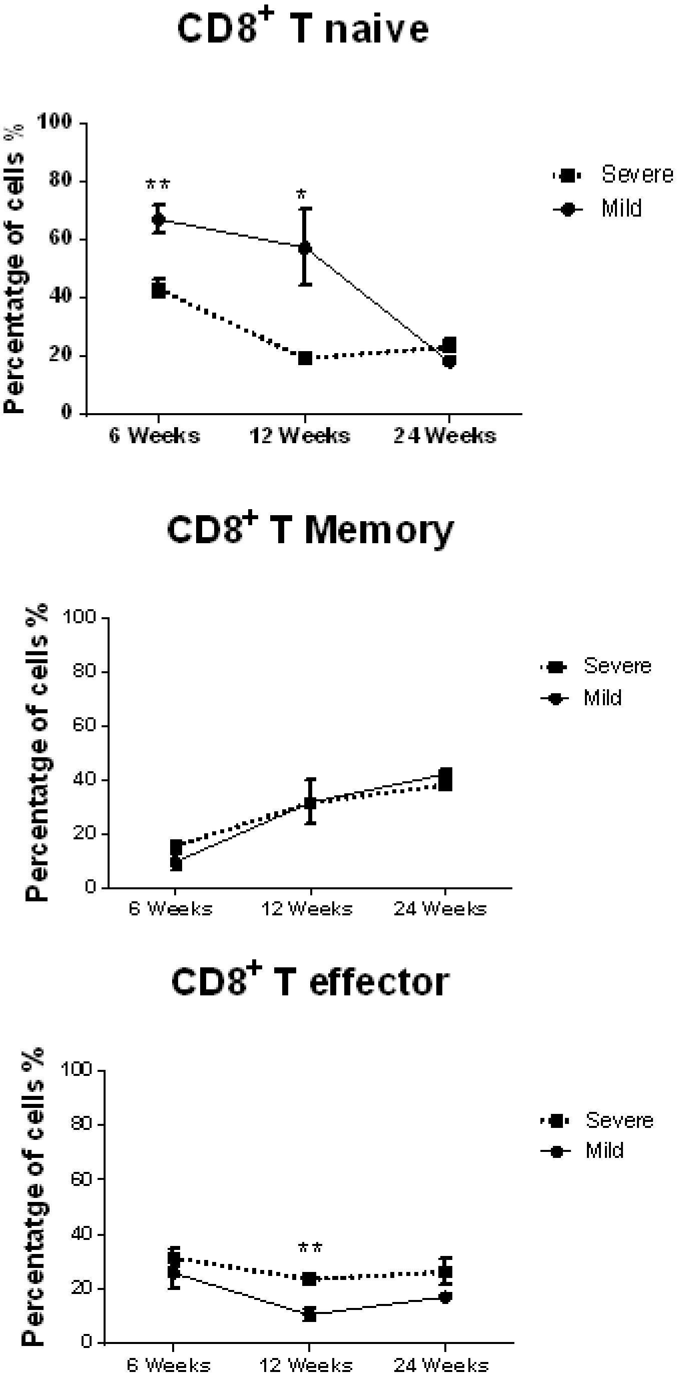

Figure S10. ***RORG* gene expression in SLE COVID-19 patient at week 6 obtained by RT-qPCR.** Significant difference were found between SLE patients who were completely recovered from COVID-19 infection (N=7) than patients with poor outcome (N=5).

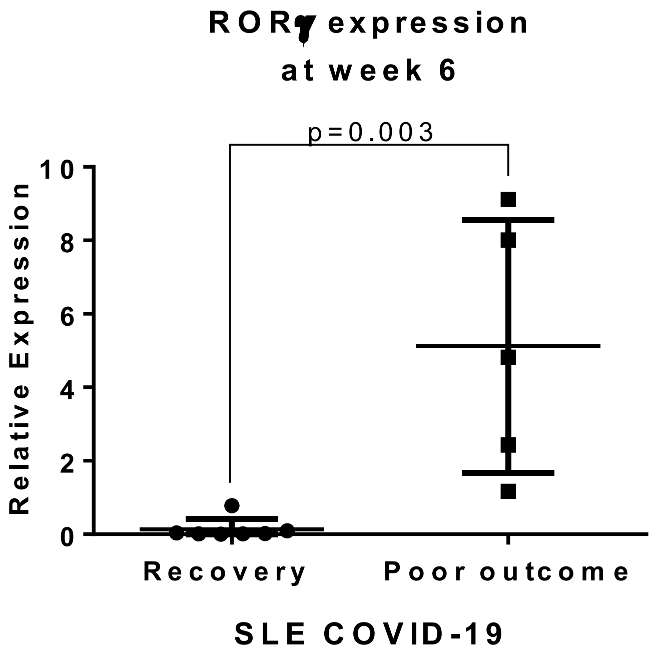

Figure S11 . Flow cytometry longitudinal interferon-gamma (IFNG) gene expression levels in convalescent COVID-19 lupus patients (n=12). A) Differences between antibody IgG produced patients (anti-RBD IgG+) and patients without antibodies (anti-RBD IgG-) were found only at 6 weeks after onset of symptoms in IFNG gene expression (p=0.0425). **B)** Not difference was found between convalescent COVID-19 lupus patients according to their severity.

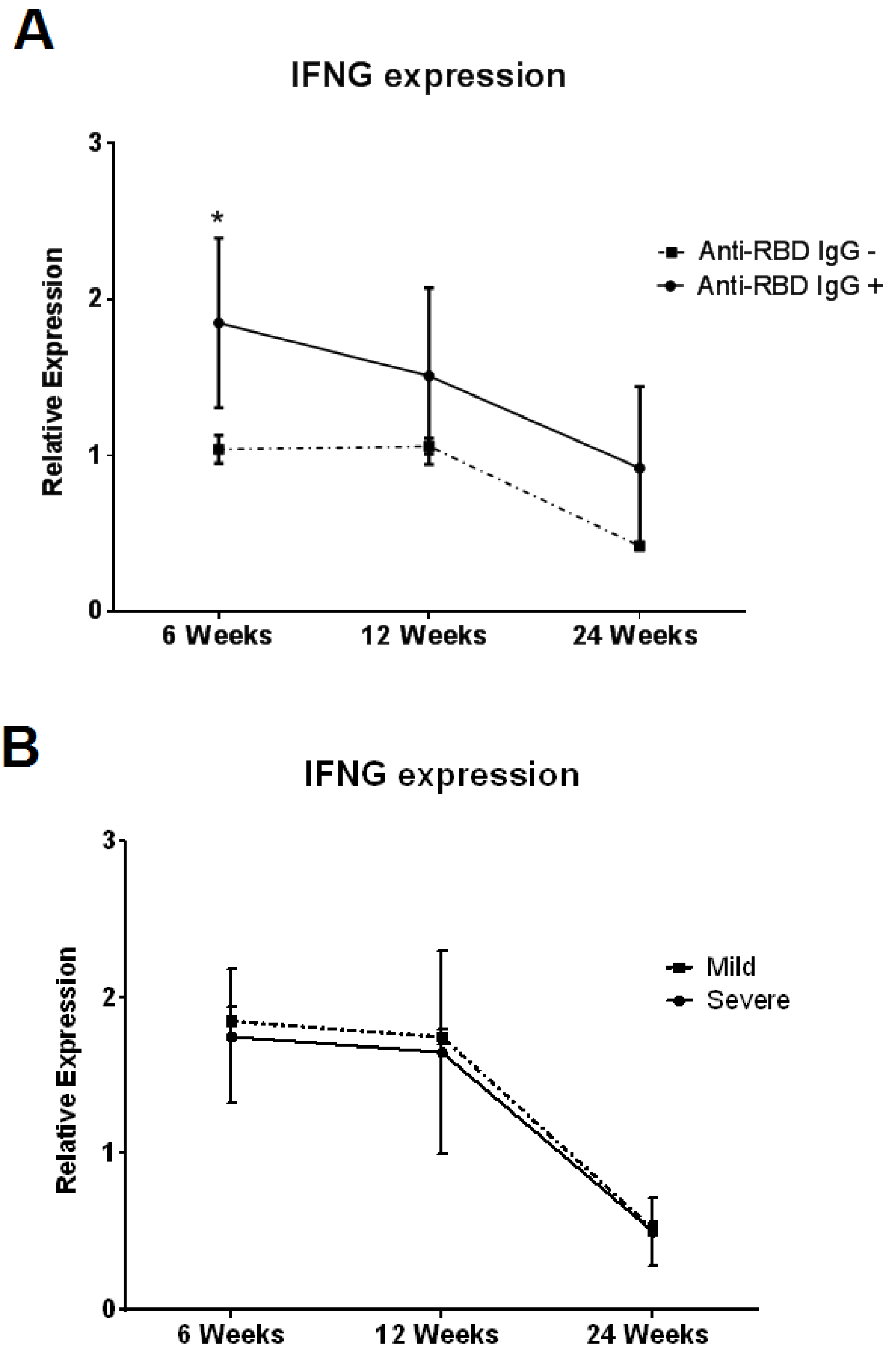

5. Supplementary Tables

Table S1. Conjugated antibodies used in Flow Cytometry analysis.

B cell subsets	Supplier	Code
**CD19**	BD Biosciences	345788
**CD27**	BD Biosciences	558664
**CD38**	BD Biosciences	555460
**IgD**	BD Biosciences	555779
**T cell subsets**	**Supplier**	**Code**
**CD3**	BD Biosciences	340662
**CD4**	BD Biosciences	561842
**CD8**	BD Biosciences	561421
**CD69**	BD Biosciences	557050
**CD197 (CCR7)**	BD Biosciences	562555
**CD45RA**	BD Biosciences	560675
**CD25**	BD Biosciences	340939
**FOXP3**	BD Biosciences	560046
**NK cell subsets**	**Supplier**	**Code**
**CD16**	BD Biosciences	561842
**CD56**	BD Biosciences	555369

Table S2. Primer IDs used in Taqman RT-qPCR from Applied Biosystems.

Gene	Assay ID (TaqMan)
**GADPH**	Hs02786624_g1
**TRIM21**	Hs00172616_m1
**INFG**	Hs00989291_m1
**IL4**	Hs00174122_m1
**IL2**	Hs00174114_m1
**IL17A**	Hs00174383_m1
**IL10**	Hs00961622_m1
**IL12A**	Hs01073447_m1
**CXCL8 (IL8)**	Hs00174103_m1
**IL6**	Hs00174131_m1
**IL1A**	Hs00174092_m1
**IL1B**	Hs01555410_m1
**TNF**	Hs00174128_m1
**IFNA1**	Hs04189288_g1
**IFNB1**	Hs01077958_s1
**OASL**	Hs07291357_m1
**MX1**	Hs00895608_m1
**ISG15**	Hs01921425_s1
**GATA3**	Hs00231122_m1
**TBX21 (T-bet)**	Hs00894392_m1
**RORC**	Hs01076112_m1
**FOXP3**	Hs01085834_m1
**OASL**	Hs07291357_m1
**MX1**	Hs00895608_m1
**ISG15**	Hs01921425_s1

Table S3. Baseline demographics and disease characteristics of COVID-19 like symptoms SLE patients.

Characteristic	COVID-19-like symptoms patients (RT-qPCR not performed, N=16)
**Women**	15 (93.75)
**Age, mean (SD), years**	46.19 (13.13)
**Healthcare professional**	0 (0.00)
**Race or ethnic group**	
**White**	14 (87.50)
**Hispanic/Latin American Origen**	2 (12.50)
**SLE disease activity**	
**SLE disease, median (range), years**	18.06 (8-26)
**Total SLEDAI-2K score, range (SD)**	1.69 (0-4)
**SLEDAI≥6**	0 (0.00)
**History of SLE clinical features**	
**Musculoskeletal**	15 (93.75)
**Mucocutaneous**	13 (81.25)
**Cardiorespiratory**	2 (12.5)
**Renal**	5 (31.25)
**Antiphospholipid syndrome**	2 (12.5)
**Leucopenia (<3x10E9)**	2 (12.5)
**Lymphopenia (<3x10E9)**	4 (25.00)
**Autoantibody status**	
**ANA titer ≥1/80**	14 (87.50)
**Anti-dsDNA antibodies ≥15 IU/mL**	8 (50.00)
**Anti-dsDNA antibodies, mean (range), IU/mL**	63.74 (3-225)
**C3 and/or C4 below lower limit of normal**	3 (18.75)
**Anti-SSA/Ro antibodies**	1 (6.25)
**Anti-SSB/La antibodies**	1 (6.25)
**Coexisting comorbidities**	
**Hypertension**	5 (31.25)
**Obesity**	1 (6.25)
**COVID-19**	
**Known COVID-19 contact**	3 (18.75)
**COVID-19 symptoms**	
**Fever**	5 (31.25)
**Shortness of breath**	5 (31.25)
**Cough**	14 (87.50)
**Anosmia/Ageusia**	2 (12.50)
**Diarrhea**	2 (12.50)
**Fatigue**	15 (93.75)
**Headache**	6 (37.50)
**Treatments**	
**Antimalarial agents**	4 (57.14)
**Serum concentration levels, mean (range), ng/mL**	1338 (503-4016)
**Immunosuppressive therapy**	5 (31.25)
**Corticosteroids use,**	11 (68.75)
**Daily prednisone dose, mean (range), mg/day**	2.81 (0-5)

Abbreviations: RT-qPCR= Reverse-transcriptase quantitative PCR; SLE= Systemic lupus erythematosus; CLE=Cutaneous Lupus Erythematosus; SLEDAI-2K= Systemic Lupus Erythematosus Disease Activity Index 2000; SLICC-DI = Systemic Lupus International Collaborating Clinics Damage Index. MMF = mycophenolate mofetil; EC-MPS = enteric-coated mycophenolate sodium. Reference ranges are as follows: anti-double-stranded DNA antibodies, <15 IU per milliliter; serum C3 (mg/dL), 85 to 110; serum C4 (mg/dL), 10 to 40.

Table S4. Multivariable logistic regression analyses were performed to obtain the relative risk in each subgroup analyses for COVID 19 infection in SLE. The values were similar to the independent analysis.

Risk factors	Relative risk in a multivariate logistic regression
HCWs	9.86 (3.82 – 25.47)
Anti-SSA/Ro52 antibodies	5.34 (2.29 – 12.42)
Higher prednisone dose (7.5 mg/day)	3.15 (0.94 – 10.58)
Immunosuppressant treatment	2.15 (0.90 – 5.16)

Table S5 . Demographic characteristics of the RT-qPCR confirmed COVID-19 and associations with SARS-CoV-2 IgG positivity

Characteristic	SARS-CoV-2 antibody positive patients (N=21)	SARS-CoV-2 antibody negative patients (N=7)	p
**Women**	20 (95.24)	6 (85.71)	0.444
**Age, mean (SD), years**	53.48 (13.51)	47.14 (10.06)	0.266
**Race or ethnic group**			
**White**	16 (76.19)	5 (71.43)	1.000
**Hispanic/Latin American Origen**	5 (23.81)	2 (28.57)	1.000
**Type of Lupus Disease**			
**Cutaneous Lupus Erythematosus (CLE)**	3 (14.29)	1 (4.76)	1.000
**Systemic Lupus Erythematosus (SLE)**	18 (85.72)	6 (85.71)	1.000
**SLE disease activity**			
**Total SLEDAI-2K score, mean (SD)**	0.81 (0-8)	1.43 (0-7)	0.696
**SLEDAI≥6**	2 (9.52)	1 (14.28)	1.000
**Autoantibody status**			
**ANA titer ≥1/80**	21 (100)	7 (100)	1.000
**Anti-dsDNA antibodies ≥15 IU/mL**	8 (38.10)	1 (14.28)	0.372
**C3 and/or C4 below lower limit of normal**	5 (23.81)	0 (0.00)	0.289
**Anti-SSA/Ro52 antibodies**	9 (42.86)	3 (42.86)	1.000
**Coexisting comorbidities**			
**Hypertension**	11 (44.0)	5 (23.81)	0.662
**Cardiovascular disease**	3 (14.28)	1 (4.76)	1.000
**Pulmonary disease**	1 (4.00)	0 (0.00)	1.000
**Diabetes**	2 (8.00)	0 (0.00)	1.000
**Obesity**	2 (8.00)	1 (4.76)	1.000
**Antiphospholipid syndrome**	7 (28.00)	4 (19.05)	0.381
**Depressive syndrome**	0 (0.00)	0 (0.00)	1.000
**Treatments**			
**Antimalarial agents**	10 (47.62)	4 (57.14)	1.000
**Immunosuppressive therapy**	9 (42.86)	5 (71.43)	0.385
**MMF/EC-MPS**	6 (28.57)	5 (71.43)	0.076
**Azathioprine**	2 (9.52)	2 (28.57)	0.253
**Tacrolimus**	3 (14.29)	3 (42.86)	0.144
**Methotrexate/leflunonide**	4 (19.05)	0 (0.00)	0.545
**Biological therapy**	3 (14.29)	1 (14.28)	1.000
**Tocilizumab**	1 (4.76)	0 (0.00)	1.000
**Etanercept**	2 (9.52)	1 (14.28)	1.000
**Corticosteroids use**	11 (52.38)	6 (85.71)	0.191
**Daily prednisone dose, mean (SD), mg/day**	2.26 (10.77)	4.64 (3.36)	0.574
**>7.5 mg/d at baseline**	1 (4.76)	3 (42.86)	0.038

* Except where indicated otherwise, values are the number (%).

Abbreviations: RT-qPCR= Reverse-transcriptase quantitative PCR; SLE= Systemic lupus erythematosus; CLE=Cutaneous Lupus Erythematosus; SLEDAI-2K= Systemic Lupus Erythematosus Disease Activity Index 2000; MMF = mycophenolate mofetil; EC-MPS = enteric-coated mycophenolate sodium. Reference ranges are as follows: anti-double-stranded DNA antibodies, <15 IU per milliliter; serum C3 (mg/dL), 85 to 110; serum C4 (mg/dL), 10 to 40.

Table S6 . Demographic and clinical data of COVID-19 convalescent lupus patients followed longitudinally.

Characteristic	SLE COVID-19 (N=12)
**Women**	11 (91.67)
**Age, mean (SD), years**	43.92 (11.67)
**Race or ethnic group,**	
**White**	9 (75)
**Asian**	0 (0)
**Hispanic/Latin American Origen**	3 (25)
**Type of Lupus Disease**	
**Cutaneous Lupus Erythematosus (CLE)**	3 (75)
**Systemic Lupus Erythematosus (SLE)**	9 (75)
**SLE disease activity**	
**Total SLEDAI-2K score, mean (SD)**	1.92 (2.50)
**SLEDAI≥6**	2 (16.67)
**Autoantibody status**	
**ANA titer ≥1/80**	11 (91.67)
**Anti-dsDNA antibodies ≥15 IU/mL**	4 (33.33)
**C3 and/or C4 below lower limit of normal**	3 (25)
**Anti-SSA/Ro antibodies**	4 (33.33)
**Coexisting comorbidities**	
**Hypertension**	4 (33.33)
**Cardiovascular disease**	0 (0)
**Pulmonary disease**	0 (0)
**Diabetes**	1 (8.33)
**Obesity**	0 (0)
**Antiphospholipid syndrome**	2 (16.67)
**Depressive syndrome**	0 (0)
**Treatments**	
**Antimalarial agents**	5 (41.67)
**Immunosuppressive therapy**	11 (91.67)
**MMF/EC-MPS**	6 (50)
**Azathioprine**	3 (25)
**Tacrolimus**	3 (25)
**Methotrexate/leflunonide**	1 (8.33)
**Biological therapy**	1 (8.33)
**Etanercept**	1 (8.33)
**Corticosteroids use, n (%)**	5 (41.67)
**Daily prednisone dose, mean (SD), mg/day**	2.5 (3.20)
**>7.5 mg/d at baseline, n (%)**	2 (16.67)
**COVID-19 parameters**	
**Positive PCR, n (%)**	12 (100)
**Positive Serology, n (%)**	8 (66.67)
**Risk of COVID, n (%)**	12 (100)
**Mild COVID, n (%)**	6 (50)
**Severe COVID, n (%)**	6 (50)
**Symptoms, n (%)**	12 (100)
**Fever**	12 (100)
**Shortness of breath**	7 (58)
**Cough**	11 (92)
**Anosmia/Ageusia**	11 (92)
**Diarrhea**	4 (33)
**Fatigue**	6 (50)
**Headache**	7 (58)
**Chest X-ray Pneumonia, n (%)**	7 (58)
**Hospital admission, n (%)**	6 (50)
**ICU admission, n (%)**	4 (33)

* Except where indicated otherwise, values are the number (%).

Abbreviations: SLE= Systemic lupus erythematosus; CLE=Cutaneous Lupus Erythematosus; SLEDAI-2K= Systemic Lupus Erythematosus Disease Activity Index 2000; MMF = mycophenolate mofetil; EC-MPS = enteric-coated mycophenolate sodium. Reference ranges are as follows: anti-double-stranded DNA antibodies, <15 IU per milliliter; serum C3 (mg/dL), 85 to 110; serum C4 (mg/dL), 10 to 40.

Table S7. *RORG* gene expression values from SLE COVID-19 patients with poor outcome at week 6. Their values were higher than the recovered SLE COVID-19 patients.

<table>
<tr><th colspan="3">RORG gene expression at week 6</th></tr>
<tr><th>Recovery SLE COVID-19
(N=7)</th><th colspan="2">SLE COVID-19 with
poor outcome
(N=5)</th></tr>
<tr><td>0,002</td><th>Thrombosis
(N=3)</th><th>Lung opacities
(N=2)</th></tr>
<tr><td>0,015</td><td>9,112</td><td>8,01</td></tr>
<tr><td>0,777</td><td>1,174</td><td rowspan="2">2,433</td></tr>
<tr><td>0,013</td><td>4,823</td></tr>
<tr><td>0,093</td><td></td><td></td></tr>
<tr><td>0,022</td><td></td><td></td></tr>
<tr><td>0,036</td><td></td><td></td></tr>
</table>
